# Ultrasound-assisted extraction of novel ancient tea polysaccharide NATP-60a: Process optimization, structural characterization, and mechanism of alleviating DSS-induced colitis in mice

**DOI:** 10.1016/j.ultsonch.2026.108005

**Published:** 2026-08-13

**Authors:** Deyong Zeng, Jianlong Wang, Junli Liu, Bichun Hu, Weiping Wang, Shuaimin Hu, Cuihong Dai, Haitian Zhao, Weihong Lu

**Affiliations:** a Department of Food Science and Engineering, School of Medicine and Health, Harbin Institute of Technology, Harbin 150000, China; b National and Local Joint Engineering Laboratory for Synthesis, Transformation and Separation of Extreme Environmental Nutrients, Harbin Institute of Technology, Harbin 150001, China; c The Intelligent Equipment Research Center for the Development of Special Medicine and Food Resources, Chongqing Research Institute of HIT, Chongqing 401120, China; d Zhengzhou Advanced Research Institute of Harbin Institute of Technology, Zhengzhou 450003, China

**Keywords:** Nanchuan ancient tea, Ultrasound-assisted extraction, Polysaccharides, Colitis, Gut microbiota

## Abstract

Plant-derived polysaccharides are considered potentially effective treatments for colitis due to their safety and anti-inflammatory properties. This study used leaves of ancient tea trees endemic to southwestern China as raw materials, established an optimized ultrasound-assisted extraction (UAE) process, subsequently isolated and purified a novel polysaccharide NATP-60a, and systematically investigated its alleviating effect on colitis induced by oral administration of dextran sulfate sodium (DSS) in mice. The results showed that under the optimal UAE process conditions, the yield of crude polysaccharide was 3.84 ± 0.15%. Subsequently, preliminary screening was performed to obtain the crude polysaccharide NATP-60, which was further purified via DEAE-52 cellulose anion-exchange chromatography to ultimately acquire the bioactive polysaccharide fraction NATP-60a. NATP-60a was a polymer with a molecular weight of 8.4 kDa. Its main chain structure was: T-β-Rhap-1 → 3-α-D-Manp-1 → 6-α-D-Manp-1 → 3,6-β-Galp-1 → 5-α-L-Araf-1 → 4-β-D-Xylp-1 → 6-β-Galp-(1 → . NATP-60a significantly alleviated DSS-induced colitis symptoms in mice, restored gut microbiota homeostasis, increased the abundance of beneficial bacteria such as *Muribaculum*, and reversed DSS-induced depletion of short-chain fatty acids. Metabolomics results showed that NATP-60a mainly corrected metabolic disorders by regulating amino acid metabolism, especially tryptophan metabolism and tyrosine metabolism. The above results demonstrate that NATP-60a improves DSS-induced colitis by modulating gut microbiota and metabolic reprogramming, making it a potential prebiotic for colitis treatment.

## Highlights

• This study optimized NATP ultrasound-assisted extraction, yielding 3.84 ± 0.15% under optimal conditions.
• A novel polysaccharide, NATP-60a, was identified and shown to significantly alleviate DSS-induced colitis in mice.
• The protective effect is mediated by reshaping gut microbiota, specifically enriching beneficial microbial taxa.
• Multi-omics analysis revealed that the mechanism involves rectifying host metabolic pathways, particularly amino acid and cofactor biosynthesis.

## Graphical abstract

**Figure:**
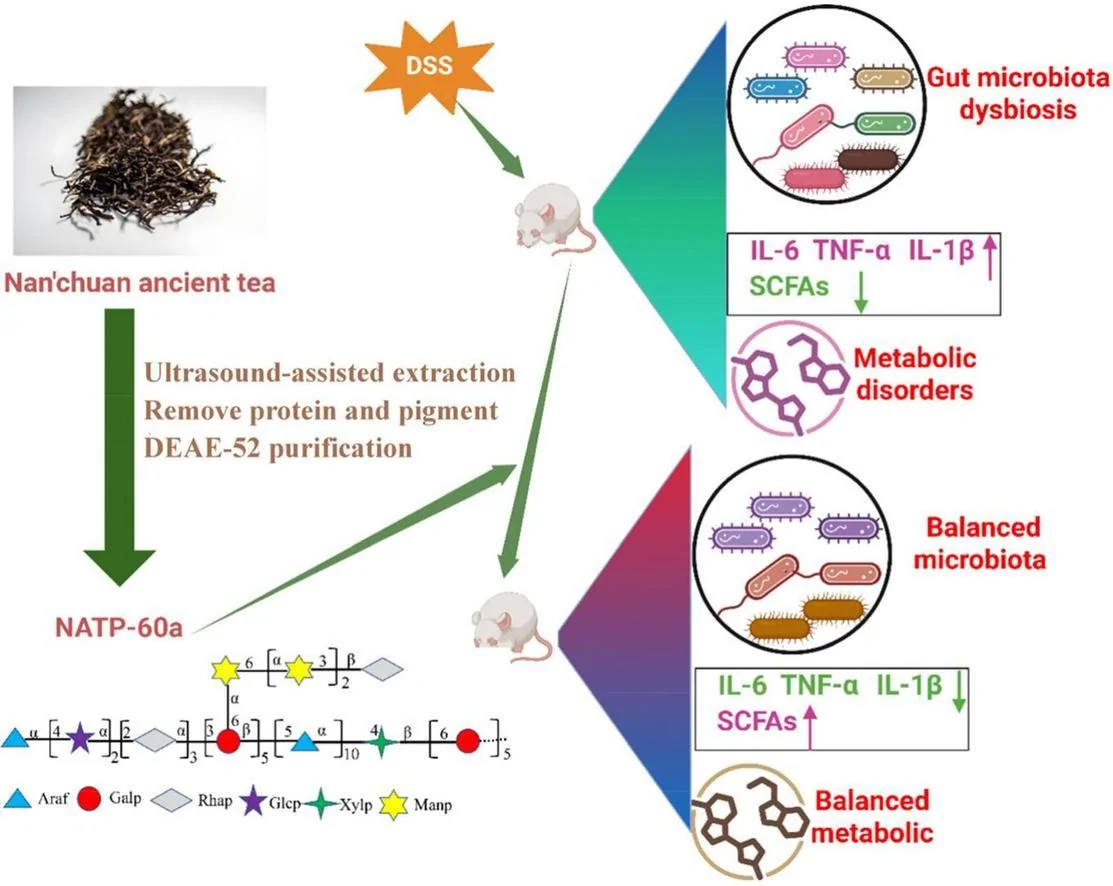

## Introduction

Inflammatory bowel disease (IBD) encompasses two main clinical entities, among which ulcerative colitis (UC) is a chronic, multifactorial intestinal inflammatory disorder [1]. Its clinical presentation is characterized by recurrent flare-ups and a protracted disease course, which significantly impairs patients' quality of life and may potentially lead to long-term complications [2]. Gut microbiota dysbiosis has been established as a key mechanism underlying the pathogenesis of colitis [3], which promotes the application of therapeutic approaches including probiotics and fecal microbiota transplantation (FMT) to remodel the microbial community and alleviate disease symptoms. However, these exogenous therapeutic approaches frequently elicit adverse reactions including abdominal distension, pain, and infections [4]. Given these limitations, developing novel strategies to safely and effectively manage colitis through modulation of the host's endogenous gut microbiota is urgently required.

Tea is the world's most consumed beverage after water and one of the three major non-alcoholic beverages. Classified as key active constituents of tea, tea polysaccharides have abundant bioactivities, involving tumor resistance, immunity adjustment, oxidation resistance, hypoglycemia relief, anti-aging and hepatic protection [5], [6], [7], [8]. Relevant recent studies illustrate that tea polysaccharides effectively suppress the emergence of AOM/ dextran sulfate sodium (DSS)-caused colitis-associated colorectal cancer in mice [9]. Fuzhuan brick tea polysaccharides alleviate symptoms in experimental colitis [10], [11]. Dahuang tea polysaccharides exert protective effects on physical intestinal barrier, which is realized through retaining mucosal completeness and promoting tight junction protein expression. It can intensify chemical barrier function by modulating glucagon-like peptide-1 abundance and expanding the population of mucin-generating goblet cells. Besides, these polysaccharides block the conversion of immune cell subgroups and cytokines into pro-inflammatory patterns to consolidate intestinal immune barrier [12].

Recent evidence indicates that tea polysaccharides act as carbon substrates for intestinal microbes, elevating microbial diversity, reshaping community structure and stimulating the synthesis of short-chain fatty acids (SCFAs), and consequently improving gut health [13]. For instance, Qingzhuan dark tea polysaccharides ameliorate colitis by increasing the contents of acetate, propionate, and butyrate, and promoting shifts in the abundance of *Bacteroidetes* and *Firmicutes* [14]. Raw polysaccharides of Tieguanyin oolong tea could counteract the growth surge of pathogenic *Helicobacter pylori*, while enhancing the relative richness of beneficial bacterial species like *Akkermansia, Lachnospiraceae* and *Odoribacter* [15]. Crude Fuzhuan brick tea polysaccharides enhanced intestinal barrier function by increasing tight junction proteins (Occludin, Claudin-1, and ZO-1) and alleviated colitis by promoting the growth of probiotics like *Bacteroides, Parasutterella,* and *Collinsella.* Clearly, tea polysaccharides are a strong candidate as a dietary supplement or therapeutic agent for alleviating colitis, offering advantages such as cost-effectiveness and accessibility compared to polysaccharides from other sources [16].

Nanchuan ancient tea (NAT; *Camellia nanchuanica*) is a unique tea variety endemic to Nanchuan District, Chongqing, China, and was historically renowned as a “Tribute Tea”. It possesses significant research value due to its unique growing environment and genetic characteristics. While preliminary studies have investigated the sensory quality and aroma components of NAT, research into its polysaccharides remains unreported. Ultrasound-assisted extraction (UAE) can rapidly fracture plant cell wall structures and facilitate polysaccharide outflow via cavitation, mechanical vibration, and thermal effects. In contrast to conventional hot water extraction (HWE), UAE exhibits prominent advantages including high extraction efficiency, low energy consumption, environmental friendliness, high extract purity, and wide applicability, and has become one of the most promising green and efficient technologies in the field of natural active polysaccharide extraction [17]. Wang et al. reported that UAE improved the extraction yield and biological activity of Hawk Tea polysaccharides compared with HWE [18]. To date, the application of UAE to the extraction of NAT polysaccharides (NATP) has not been reported.

In this study, we established a UAE process for the mature leaves of NAT to obtain NATP, elucidated the fine structure of NATP, and explored its ability to alleviate colitis as well as the underlying mechanisms using a DSS-induced murine colitis model. This investigation supplies theoretical support for the subsequent exploitation and popularization of NATP in intestinal protection and colitis relief. It will also broaden the pathway for promoting the in-depth processing and high-value utilization of NAT in Chongqing.

## 2 Materials and methods

### 2.1 Materials

NAT leaves were purchased locally in Nanchuan, Chongqing, China.

### 2.2 Ultrasonic-assisted extraction process optimization for NATP

In accordance with the procedure reported by Wang et al. [18], the NAT raw material was first subjected to defatting treatment. Following initial single-factor screening experiments, we identified three critical process variables for subsequent optimization: ultrasonic power, extraction duration, and extraction temperature. Ultrasonic power was set at 250 W, 300 W, and 350 W; ultrasonic extraction time was set at 55 min, 75 min, and 95 min; and ‘extraction temperature was set at 45 °C, 55 °C, and 65 °C. Subsequently, response surface methodology (RSM) with Box-Behnken design (BBD) was conducted to optimize the key parameters of the extraction process.

### 2.3 Isolation and purification of NATP

The extracts obtained under the optimal UAE conditions in Section 2.2 were combined, followed by filtration and centrifugation. The clarified liquid phase was concentrated, then underwent processes to remove proteins and colorants, and was dialyzed (3.5 kDa MWCO) [19]. After concentrating the dialyzed solution, sequential ethanol precipitation was performed using concentrations of 30 %, 60 %, and 80 %. Each precipitate was isolated by centrifugation and lyophilized, yielding distinct crude NATP fractions. The fraction obtained with 60 % ethanol underwent further purification via DEAE-52 chromatography, resulting in a refined polysaccharide designated NATP-60a.

### 2.4 Chemical composition analysis of NATP-60a

The total carbohydrate content of NATP-60a was determined by the phenol–sulfuric acid method using galactose as the standard, with absorbance detected at 490 nm. The protein content was measured by the Coomassie brilliant blue method with bovine serum albumin (BSA) as the standard, and absorbance was recorded at 595 nm.

### 2.5 Molecular weight and monosaccharide composition of NATP-60a

NATP-60a solution at 1 mg/mL was analyzed via HPGPC equipped with UltraHydrogel™ column (7.8 × 300 mm, Waters, Japan) [20]. PBS (pH 6.8) was used for isocratic elution at 1 mL/min. Molecular mass determination utilized a pre-established calibration curve.

For monosaccharide composition analysis, 10 mg NATP-60a was hydrolyzed with 2.0 mL 2.0 mol/L trifluoroacetic acid at 105 °C for 4 h. Post-hydrolysis, residual trifluoroacetic acid was evaporated under reduced pressure. The resulting hydrolysate was reconstituted in 300 µL deionized water. Subsequent PMP derivatization involved the sequential addition of NaOH solution and methanolic 1-phenyl-3-methyl-5-pyrazolone. Once the derivatization reaction was complete, chloroform extraction was performed prior to high-performance liquid chromatography analysis [21].

### 2.6 UV–Vis and FT-IR spectroscopic analysis

For UV–Vis measurement, NATP-60a was dissolved in ultrapure water to a concentration of 0.5 mg/mL. The absorption spectrum was recorded in the range of 200–800 nm using a UV–Vis spectrophotometer. For FT-IR measurement, dried NATP-60a powder was thoroughly ground with anhydrous KBr and compressed into a transparent pellet. The IR spectrum was collected in the wavenumber range of 4000–500 cm^−1^ [22].

### 2.7 NATP-60a structural analysis

#### 2.7.1 Methylation experiment

NATP-60a was dissolved in dimethyl sulfoxide (DMSO), and methylated with methyl iodide in the presence of sodium hydroxide under a nitrogen atmosphere. After quenching and extraction, the methylated product was hydrolyzed with trifluoroacetic acid, reduced to alditols using sodium borodeuteride (NaBD_4_), and acetylated with acetic anhydride to form partially methylated alditol acetates (PMAAs). The final derivatives were analyzed by gas chromatography-mass spectrometry (GC–MS) [23].

#### 2.7.2 NMR experiment

Briefly, NATP-60a was dissolved in D_2_O and transferred to an NMR tube for 1D (^13^C and ^1^H) and 2D (HSQC, COSY, HMBC) spectral detection using a 600 MHz NMR spectrometer.

### 2.8 Microstructural characterization

Scanning electron microscopy (SEM) was performed using a ZEISS field‑emission scanning electron microscope (ZEISS, Germany). Samples were gold-sputtered prior to imaging. For atomic force microscopy (AFM), NATP-60a aqueous solutions were deposited onto mica substrates, air-dried, and chemically fixed before analysis using a Dimension Fastscan system (Bruker, Germany). The crystalline structure was evaluated via powder X-ray diffraction analysis.

### 2.9 In vitro anti-inflammatory activity evaluation

RAW 264.7 macrophages were cultured in standard DMEM medium. NATP-30, NATP-60 and NATP-80 fractions were tested for cytotoxicity via the CCK-8 assay. For anti-inflammatory assays, cells were pretreated with polysaccharides then stimulated by LPS, and supernatant levels of NO, TNF-α, IL-1β and IL-6 were quantified with commercial kits.

### 2.10 Animals and treatment

Male Kunming mice (5 weeks old) were randomized into four groups (n = 8/group): normal control (CK), colitis model (MC), NATP-60a intervention (NATP-60a), and positive control (YC). All animal experiments were performed in strict accordance with the National Guide for the Care and Use of Laboratory Animals. Following one-week acclimatization, the MC, NATP-60a, and YC groups received 4% DSS ad libitum in drinking water to induce colitis, while CK consumed normal water. Daily intragastric administrations were performed for 14 consecutive days: NATP-60a received 400 mg/kg·bw NATP-60a (the optimal high therapeutic dose determined via pilot dose screening and established experimental paradigm in the field), YC obtained 100 mg/kg·bw 5-aminosalicylic acid, while CK and MC were administered physiological saline. Body mass was monitored throughout the experimental period. Twenty-four hours post-final treatment, subjects were weighed, euthanized, and subjected to necropsy. Colonic and cecal tissues were immediately harvested; colonic dimensions were documented photographically for length quantification.

### 2.11 Determination of inflammatory mediators and cytokines in colon tissues

The levels of pro-inflammatory cytokines (TNF-α, IL-1β, and IL-6) in colon tissues were measured using enzyme-linked immunosorbent assay (ELISA) kits according to the manufacturer’s instructions. Nitric oxide (NO) content was detected via a colorimetric nitrate reductase assay kit based on the Griess reaction principle.

### 2.12 Histological analysis

Mouse colons were formalin-fixed, paraffin-embedded, sectioned, and stained for microscopy.

### 2.13 Fecal SCFA assay

Briefly, 200 mg of fecal sample was mixed with NaOH solution containing 2-methylbutyric acid as the internal standard, heated, cooled to room temperature, and incubated at 4 °C. After centrifugation, the supernatant was derivatized with isopropyl alcohol–pyridine mixture and analyzed by GC–MS [19].

### 2.14 Mouse gut microbiota analysis

Following the method described by Ren et al. [24], we performed 16S rRNA gene sequencing analysis of the mouse intestinal microbiota. DNA was extracted from mouse feces, and the 16S rRNA V4 region was amplified to construct a sequencing library and sequenced. Sequencing work was completed at Southwest Lou Biotechnology Co., Ltd. (Shanghai, China).

### 2.15 Mouse fecal metabolomics analysis

Fecal samples were homogenized and centrifuged in methanol. The supernatant was filtered with 0.22 μm membrane and stored at − 80 °C for later analysis. Metabolic profiling was performed by HPLC-MS on a Q Exactive Plus system with an ACQUITY UPLC BEH C18 column (2.1 × 50 mm, 1.7 μm; Waters, Ireland) at 30 °C. The mobile phase was 0.1% formic acid in water (A) and acetonitrile (B) at 0.30 mL/min, with a gradient program: 5% B for 2 min, linear increase to 95% B over 14 min, and re-equilibration at 5% B for 2 min. MS detection used an ESI source in both positive and negative modes (spray voltage: 3.0 kV). Full-scan MS data were acquired at 70,000 resolution (AGC target: 3 × 10⁶, max injection time: 100 ms), and tandem MS spectra were collected at 17,500 resolution with 30 eV normalized collision energy. Sheath gas voltage was 30 V for both modes, and the mass scan range was *m*/*z* 100–1000. Differential metabolites were identified using MetaboAnalyst 5.0 with thresholds of VIP > 1, fold change > 1.2, and *p* < 0.05, followed by KEGG enrichment analysis.

## 3 Results

### 3.1 Optimization of UAE of NATP

Table 1 presents the design scheme and corresponding experimental results of the NATP response surface test. On the basis of Box-Behnken design, Design-Expert 13 software was used for multiple regression analysis and model construction, and a quadratic polynomial fitting equation between NATP extraction yield and various extraction influencing factors was established as follows:

**Table 1:** Experimental design and the results.

Run	Factor 1	Factor 2	Factor 3	Yield (%)
	A:Power (W)	B:Time (min)	C:Temperature (°C)	
1	250	95	55	2.93
2	350	95	55	3.76
3	300	95	45	3.51
4	250	75	65	2.92
5	300	55	65	2.77
6	300	75	55	3.62
7	300	75	55	3.77
8	350	75	45	3.64
9	350	55	55	3.06
10	250	55	55	2.89
11	350	75	65	3.25
12	300	95	65	3.53
13	300	75	55	3.70
14	300	75	55	3.81
15	300	75	55	3.78
16	250	75	45	2.98
17	300	55	45	3.20

Yield = 3.74 + 0.2487A + 0.2262B − 0.1075C + 0.1650AB − 0.0825AC + 0.1125BC − 0.3155A^2^ − 0.2605B^2^ − 0.2230C^2^.

**Fig. 1:**
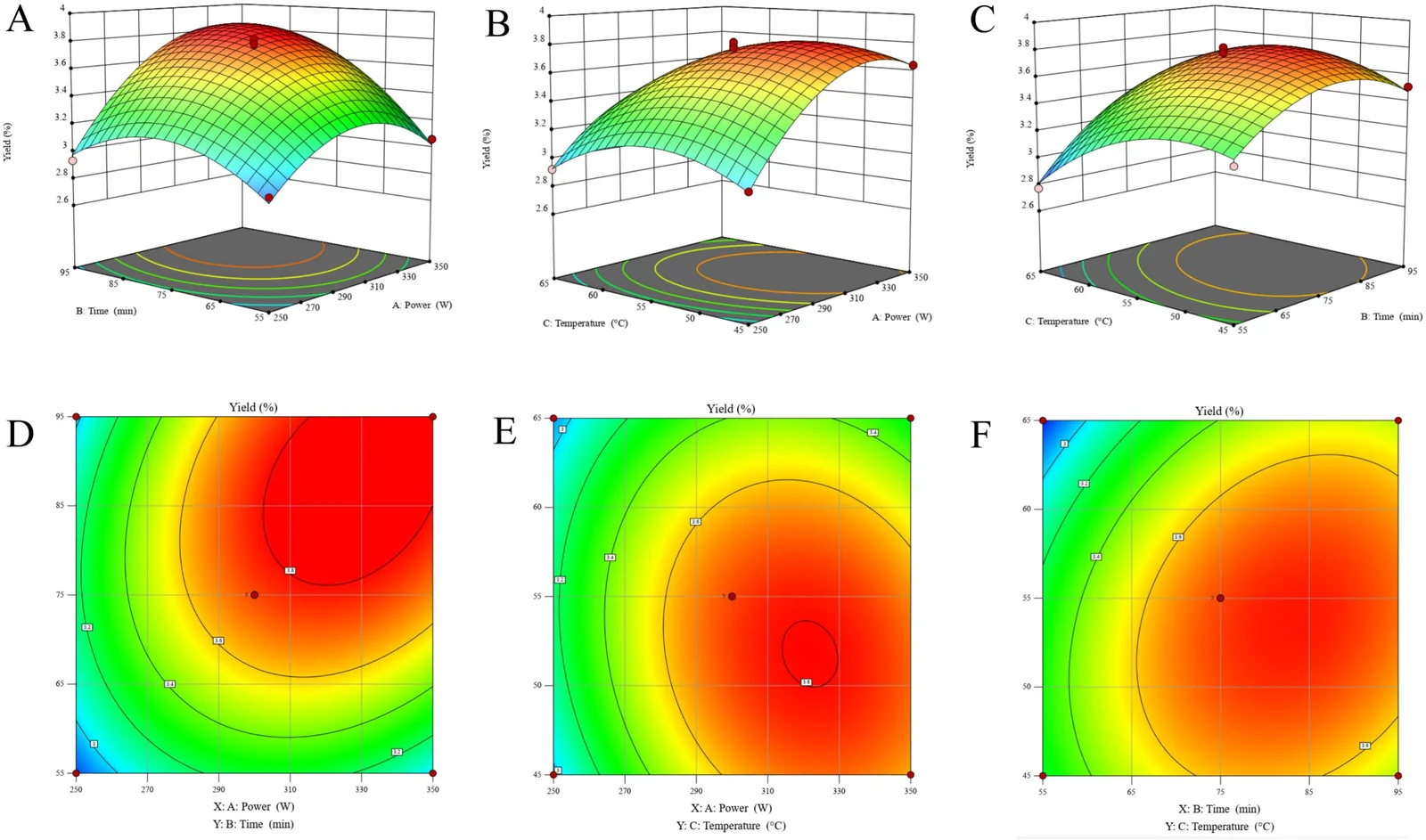
Three-dimensional (3D) response surface plots and corresponding contour plots showing the interactive effects of pairwise independent variables on the yield. (A, D): Power and Time; (B, E): Power and Temperature; (C, F): Time and Temperature.

As presented in Table 2, the regression model exhibited a coefficient of R^2^ of 0.9834 and an R^2^_adj_ of 0.9621, with a global P-value < 0.001, demonstrating that the model was highly significant. The lack-of-fit term yielded a P-value of 0.5591 (> 0.05), indicating that the lack-of-fit was not statistically significant. These results confirm that the regression model had a good fit, low experimental error, and high prediction accuracy, and can be used for the accurate prediction of NATP extraction yield and the optimization of process parameters [25], [26], [27]. The results showed that the three single factors, namely ultrasonic power (A), ultrasonic time (B), and ultrasonic temperature (C), as well as their quadratic terms A^2^, B^2^, and C^2^, all had highly significant effects on the NATP yield (P < 0.01) [28], [29]. The response surface plots shown in Fig. 1 can intuitively reflect the effects of the interactions between two factors on the polysaccharide yield. Among them, the surface slope of Fig. 1A is the steepest, which, combined with the ANOVA results in Table 2, further confirms that the interaction between ultrasonic power (A) and ultrasonic time (B) has the most significant effect on the NATP yield. Based on the response surface model analysis, the theoretical optimal extraction conditions for NATP were obtained as follows: ultrasonic power of 326.14 W, ultrasonic time of 80.88 min, and ultrasonic temperature of 54.51 °C. Under these conditions, the theoretical polysaccharide yield predicted by the model was 3.85%. Considering the operability of actual experiments, the optimal process parameters were adjusted to: ultrasonic power of 300 W, ultrasonic time of 81 min, and ultrasonic temperature of 55 °C. Three parallel trials were carried out under optimized technological conditions, with the NATP yield reaching 3.84 ± 0.15%.

**Table 2:** ANOVA for the regression model predicting NATP extraction.

Source	Sum of Squares	df	Mean Square	F-value	P-value	Significance
Model	2.20	9	0.2447	46.12	< 0.0001	significant
A-Power	0.4950	1	0.4950	93.29	< 0.0001	
B-Time	0.4095	1	0.4095	77.17	< 0.0001	
C-Temperature	0.0924	1	0.0924	17.42	0.0042	
AB	0.1089	1	0.1089	20.52	0.0027	
AC	0.0272	1	0.0272	5.13	0.0579	
BC	0.0506	1	0.0506	9.54	0.0176	
A^2^	0.4191	1	0.4191	78.98	< 0.0001	
B^2^	0.2857	1	0.2857	53.85	0.0002	
C^2^	0.2094	1	0.2094	39.46	0.0004	
Residual	0.0371	7	0.0053			
Lack of Fit	0.0138	3	0.0046	0.7905	0.5591	not significant
Pure Error	0.0233	4	0.0058			
Cor Total	2.24	16				
R^2^ = 0.9834		R^2^_Adj_ = 0.9621		C.V. = 2.17	AP = 17.7869

### 3.2 NATP-60a fragments isolation

**Fig. 2:**
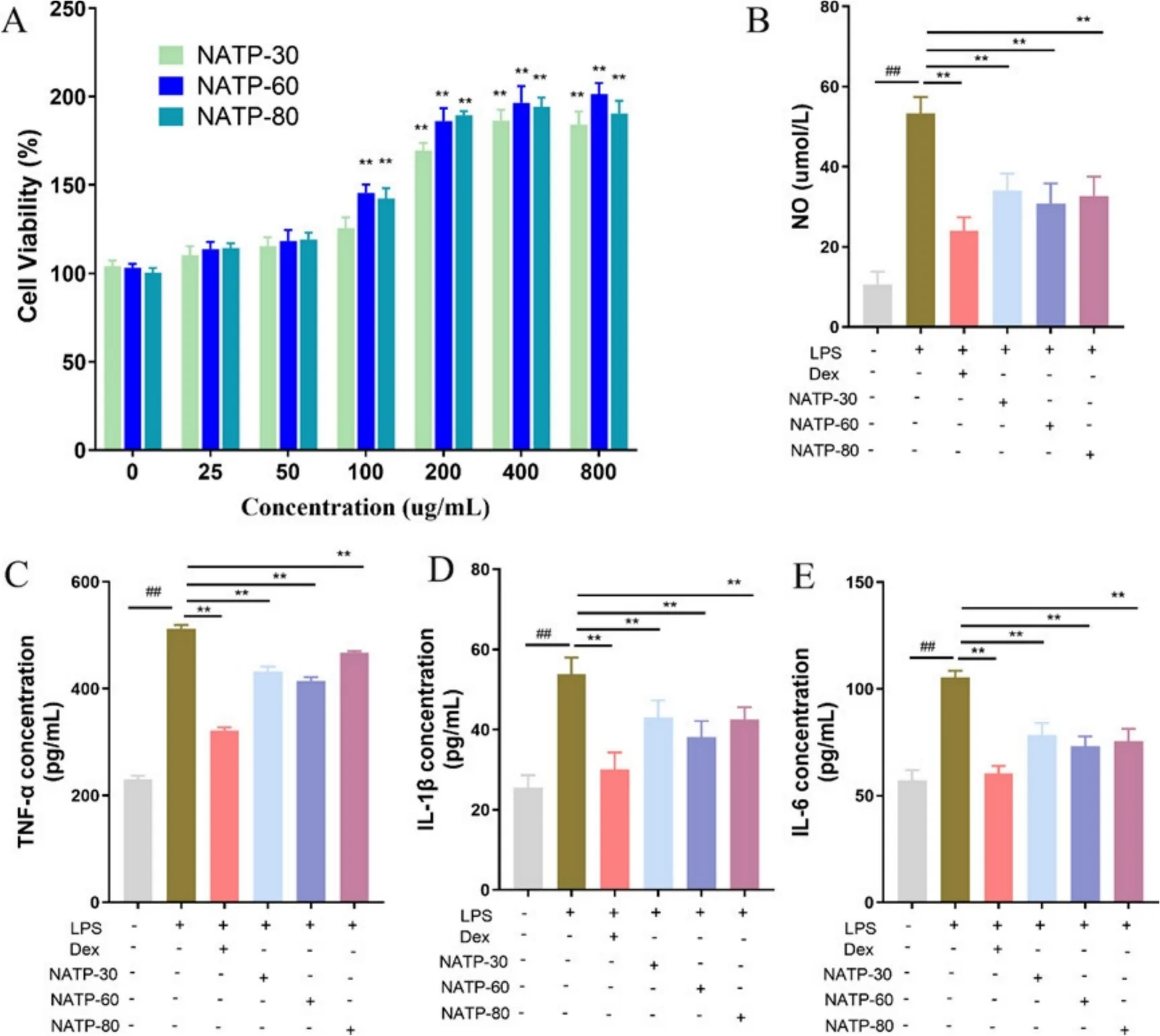
Evaluation of the activity of different fractions of NATP using RAW264.7 cells (A) The CCK8 assay using NATP (0–800 μg/mL) on RAW264.7 cells. Effects of NATP on the production of NO (B), TNF-α (C), IL-6 (D), and IL-1β (E)in the presence of 1 μg/mL LPS in RAW264.7 cells. All data were presented as mean ± SD (n = 3). ** and * p < 0.01, p < 0.05 compared with the LPS group; ^#^ and ^##^ p < 0.05, p < 0.01 compared with the control group.

**Fig. 3:**
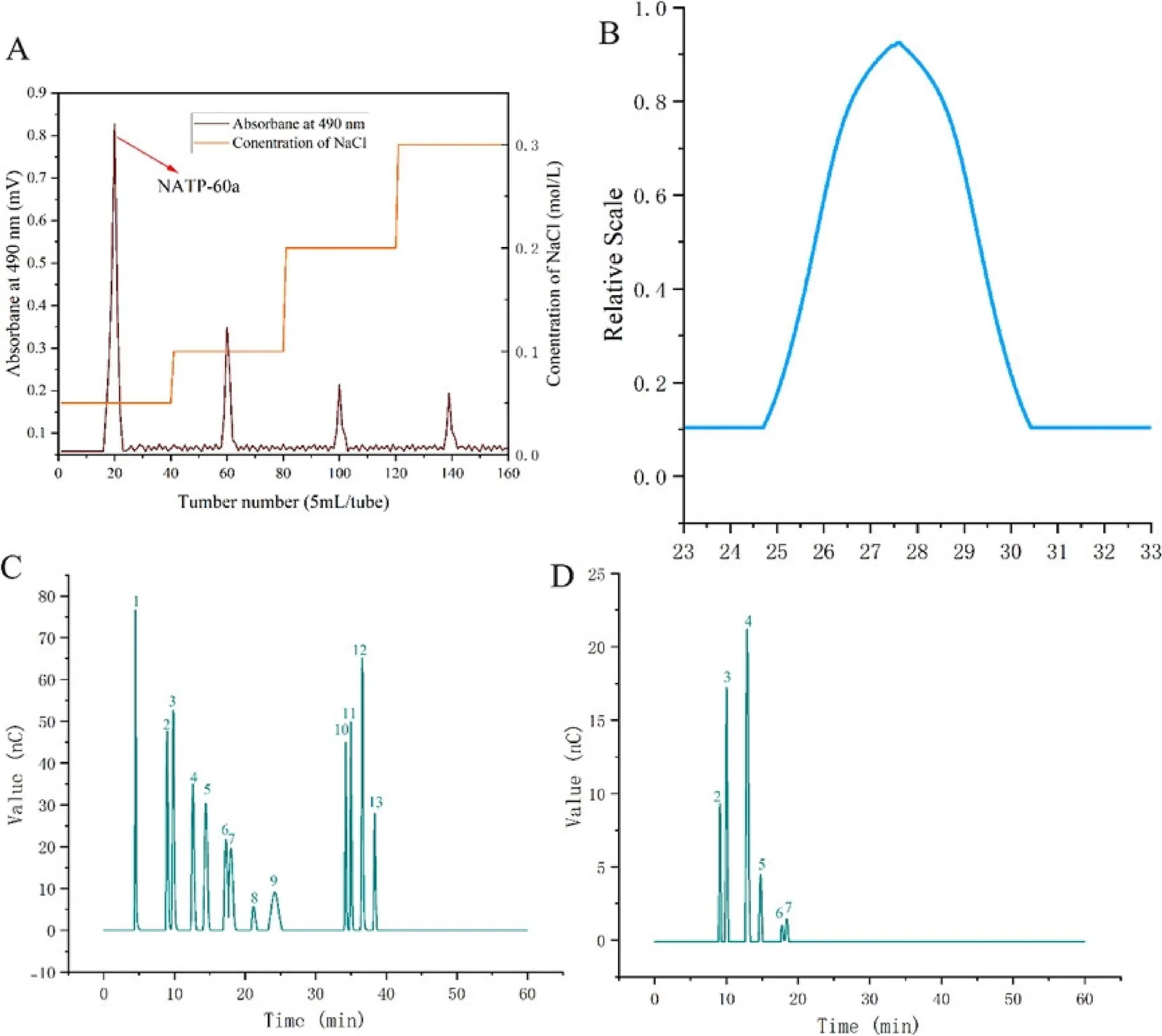
Purification, molecular weight, and monosaccharide composition analysis. A, gradient elution curve of the crude polysaccharide on a DEAE-52 column; B, purity and molecular weight determination; C, HPLC diagram of monosaccharide standards; D, HPLC diagram of the monosaccharide composition of NATP-60a. 1: Fuc: Fucose; 2: Rha: Rhamnose; 3: Ara: arabinose; 4: Gal: galactose; 5: Glc: glucose; 6: Xyl: xylose; 7: Man: mannose; 8: Fru: Fructose; 9: Rib: Ribose; 10: Gal-UA: Galacturonic Acid; 11: Glc-UA: Glucuronic Acid; 12: Man-UA: Mannuronic Acid; 13: Gul-UA: Guluronic Acid.

Three polysaccharide fractions (NATP-30, NATP-60, and NATP-80) were isolated from NAT via stepwise ethanol precipitation (30%, 60%, and 80% saturation). Preliminary screening using RAW 264.7 macrophages revealed that all fractions stimulated cellular proliferation in a concentration-dependent manner (Fig. 2A). Concurrently, each polysaccharide attenuated LPS-induced secretion of inflammatory mediators and cytokines; however, NATP-60 demonstrated significantly greater anti-inflammatory efficacy than NATP-30 and NATP-80 (Fig. 2B–D). NATP-60 was further purified via DEAE-52 anion-exchange chromatography. The major 0.1 mol/L NaCl-eluted polysaccharide fraction (Fig. 3A) was designated NATP-60a.

### 3.3 Physicochemical properties of NATP-60a

Chemical composition assays indicated that NATP-60a possessed a total carbohydrate content of 93.38% and a protein content of 0.13% (Table 3). The results showed that NATP-60a was composed of rhamnose (Rha), arabinose (Ara), galactose (Gal), glucose (Glc), xylose (Xyl), and mannose (Man) (Fig. 3C, D). We analyzed the molecular weight of NATP-60a using GPC, which revealed a relative molecular weight of 8.4 kDa (Fig. 3B). This is the first time that a polysaccharide has been isolated and identified in NAT.

**Table 3:** Component characterization of NATP-60a.

Index	NATP-60a
Total sugar content (%)	93.38 ± 2.07
Protein content (%)	0.13 ± 0.06

### 3.4 UV–Vis and FT-IR analysis

The UV–Vis spectrum of NATP-60a was scanned in the range of 200–800 nm (Fig. S1A). No distinct characteristic absorption peaks corresponding to nucleic acid (260 nm) and protein (280 nm) were detected, indicating trace amounts of protein and nucleic acid impurities in NATP-60a, which was consistent with the quantitative data in Table 3. The FT-IR spectrum (Fig. S1B) displayed typical polysaccharide absorption features. The broad band at 3411 cm^−1^ and the weak peak at 2940 cm^−1^ were attributed to O–H and C–H stretching vibrations, respectively. The absorption band at 1595 cm^−1^ was assigned to the O–H bending vibration of bound water, while the peaks at 1422 cm^−1^ and 1354 cm^−1^ were assigned to C–H bending and C–OH bending vibrations, respectively.

### 3.5 Structure characterization of NATP-60a

Methylation analysis of NATP-60a revealed the presence of ten distinct glycosidic linkage types, as summarized in Table 4. These include terminal-linked Araf (T-Araf), 1,6-linked Galp, 1,3,6-linked Galp, terminal-linked Rhap (T-Rhap), 1,2,4-linked Rhap, 1,4-linked Glcp, 1,4-linked Xylp, 1,6-linked Manp, 1,5-linked Araf, and 1,3-linked Manp, with molar ratios of 2.92: 20.49: 16.99: 3.08: 9.89: 5.78: 2.96: 3.22: 28.71: 5.96, respectively. Among these, 1,6-linked Galp and 1,5-linked Araf, were identified as the predominant linkages.

**Table 4:** The methylation analysis of NATP-60a using GC–MS.

PMAA	Linkage type	RT (min)	Major mass fragments (*m*/*z*)	Molar Ratio (%)
1,4-di-O-acetyl-2,3,5-tri-O-methyl arabinitol	T- Araf- (1→	7.97	71,87,102,118,129,145,161	2.92
1,5,6-tri-O-acetyl-2,3,4-tri-O-methyl galactitol	→6)-Galp-(1→	20.30	87,99,102,118,129,162,189,233	20.49
1,3,5,6-tetra-O-acetyl-2,4-di-O-methyl galactitol	→3,6)- Galp-(1→	24.38	87,101,118,129,189,202,234	16.99
1,5-di-O-acetyl-6-deoxy-2,3,4-tri-O-methyl rhamnitol	T- Rhap- (1→	8.63	59,72,89,102,115,118,131,145,162,175	3.08
1,2,4,5-tetra-O-acetyl-6-deoxy-3-O-methyl rhamnitol	→2,4)-Rhap-(1→	17.54	88,101,117,130,143,190,203	9.89
1,4,5-tri-O-acetyl-2,3,6-tri-O-methyl glucitol	→4)- Glcp-(1→	18.21	87,102,113,118,129,162,233	5.78
1,4,5-tri-O-acetyl-2,3-di-O-methyl xylitol	→4)- Xylp-(1→	14.28	87,102,118,129,162,189	2.96
1,5,6-tri-O-acetyl-2,3,4-tri-O-methyl mannitol	→6) −Manp-(1→	18.53	87,99,102,118,129,162,189,233	3.22
1,4,5-tri-O-acetyl-2,3-di-O-methyl arabinitol	→5)-Araf-(1→	13.76	87,102,118,129,162,189	28.71
1,3,5-tri-O-acetyl-2,4,6-tri-O-methyl mannitol	→3) −Manp-(1→	16.63	87,101,118,129,161,202,234	5.96

### 3.6 NMR spectroscopy analysis

**Fig. 4:**
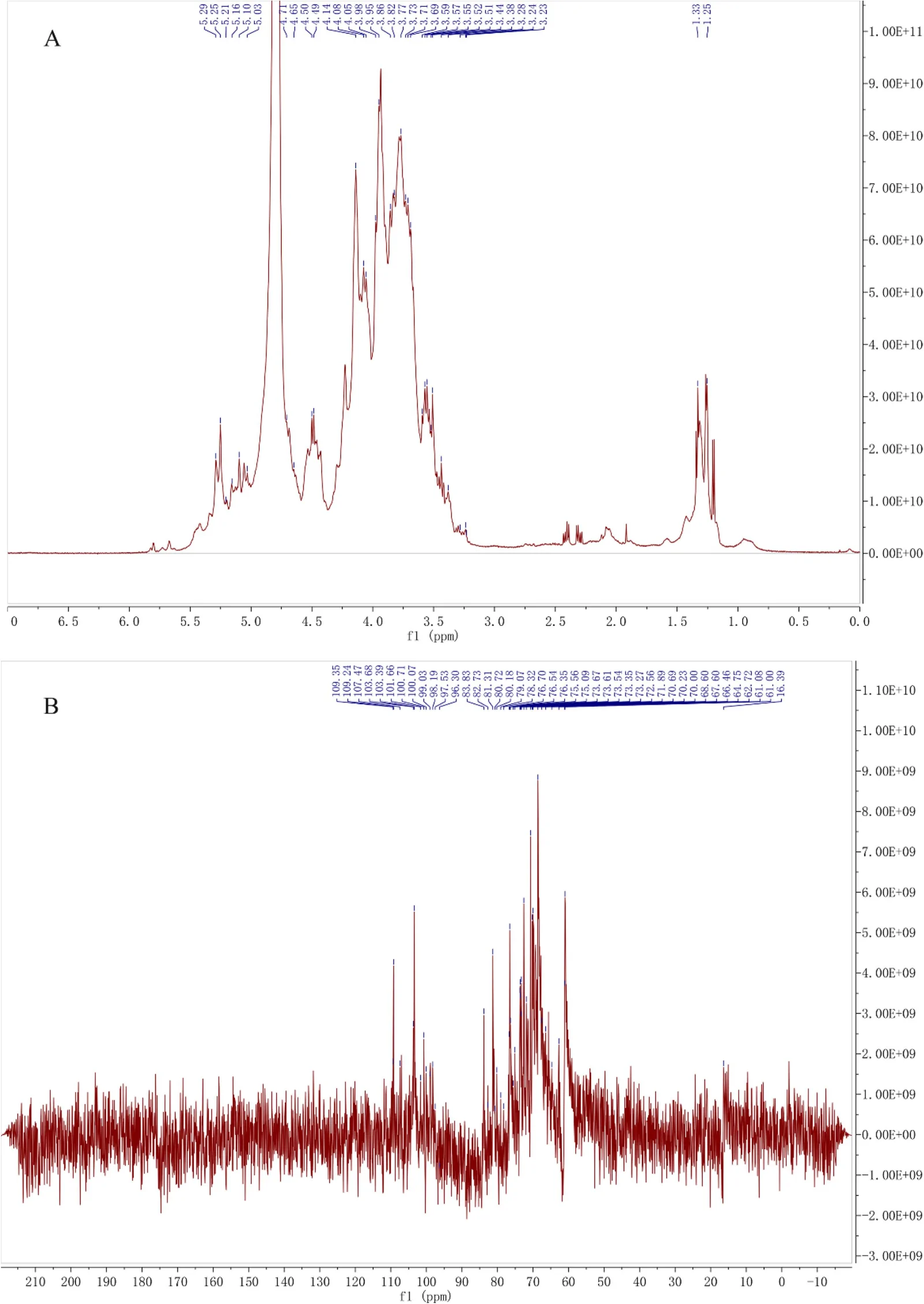
^1^D NMR characterisation of NATP-60a. (A) ^1^H NMR spectrum. (B) ^13^C NMR spectrum.

The structural features of NATP-60a were analyzed using 1D and 2D NMR spectroscopy (Fig. 4). Assignment of ^1^H and ^13^C NMR resonances in the 2D spectra was performed based on established methods [30], [31]. It is well documented that anomeric proton chemical shifts below 5.0 ppm are characteristic of β-glycosidic configurations, while those above 5.0 ppm suggest α-configurations. The ^1^H NMR spectrum of NATP-60a (Fig. 4A) revealed signals in both regions, confirming the presence of both α- and β-type glycosidic linkages.

Furthermore, the ^13^C NMR spectrum exhibited ten distinct anomeric carbon signals within the range of *δ* 96.30 to 109.24 ppm, specifically observed at *δ* 107.47, 103.39, 103.68, 96.30, 98.19, 97.53, 100.71, 100.07, 109.24, and 101.56 (Fig. 4B). This indicates the existence of ten glycosidic linkages in NATP-60a, a finding consistent with the glycosidic bond profile determined by methylation analysis.

**Fig. 5:**
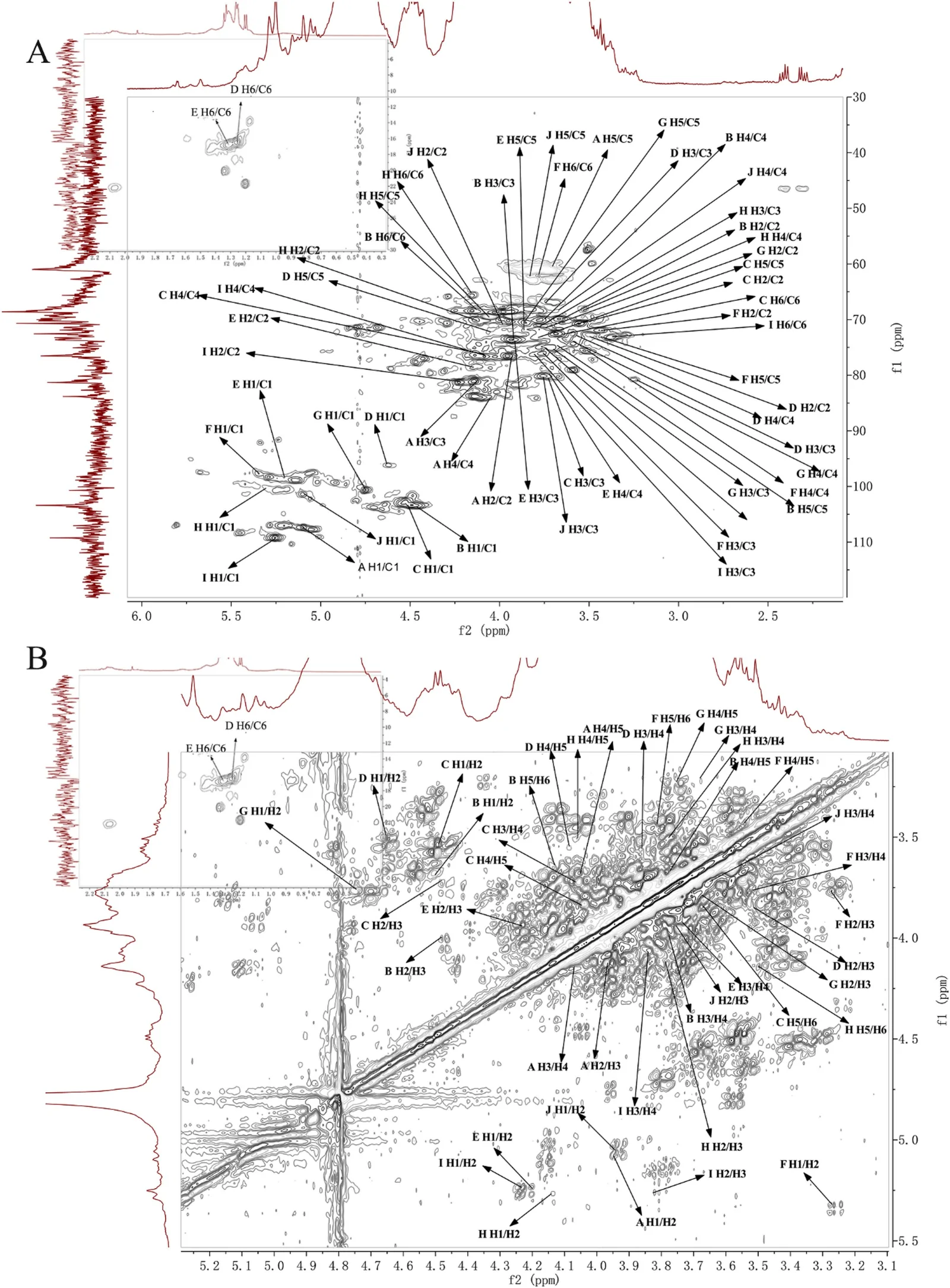

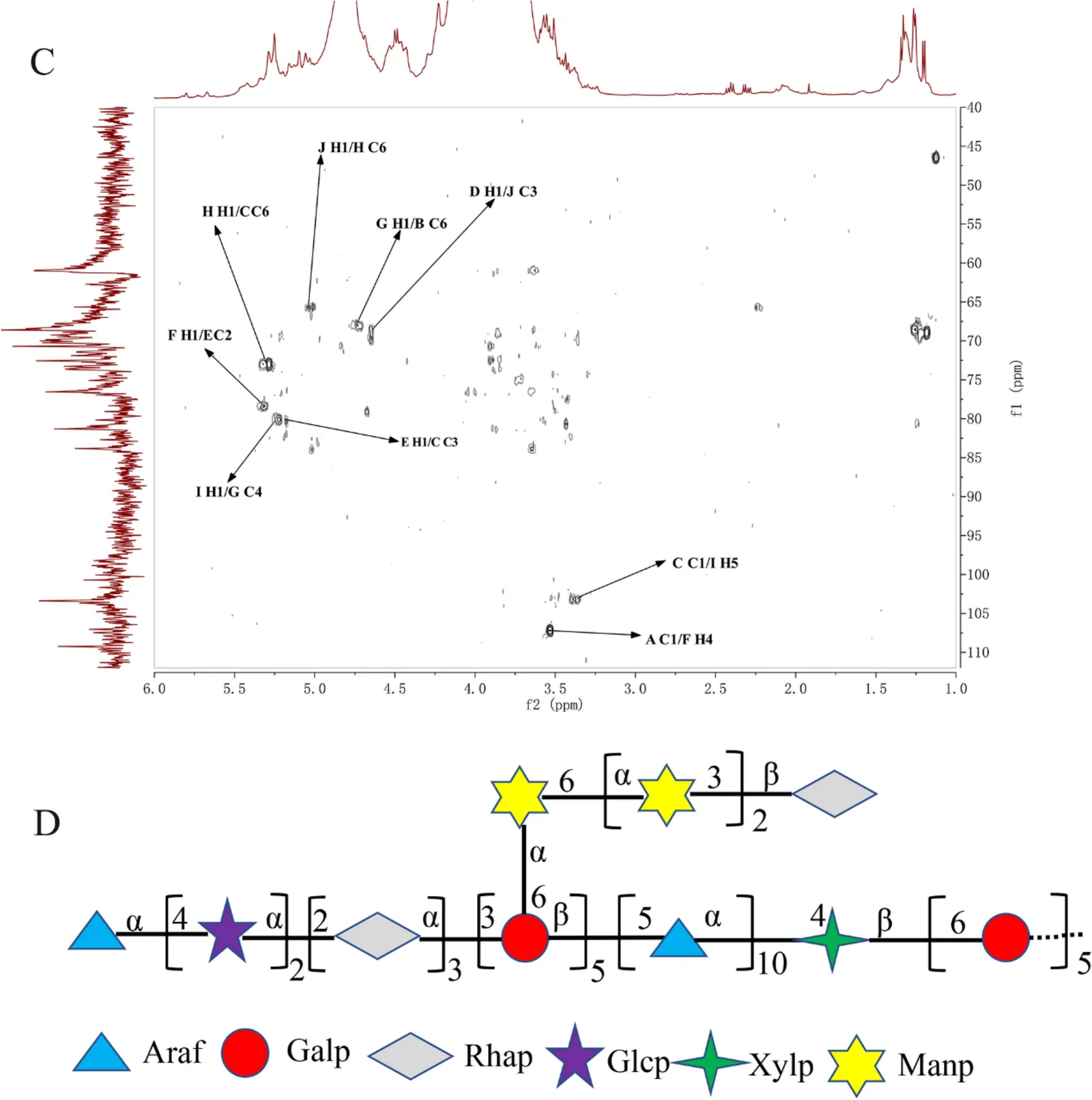
^2^D NMR spectra of NATP-60a in D_2_O. (A) HSQC spectrum. (B) COSY spectrum. (C) HMBC spectrum. (D) The putative structure of NATP-60a.

As illustrated in the HSQC spectrum (Fig. 5A), ten distinct cross-peaks were observed in the anomeric region, with chemical shifts at 5.10/107.47, 4.49/103.39, 4.50/103.68, 4.65/96.30, 5.20/98.19, 5.34/97.53, 4.74/100.71, 5.29/100.07, 5.25/109.24 and 5.05/101.56 ppm. These signals confirm the presence of ten sugar residues within NATP-60a, tentatively labeled as A through J. Subsequent structural assignment of these residues was conducted through comparative analysis with existing literature and supported by COSY and HSQC spectral data (Fig. 5A, B) [32], [33], [34], [35], [36], [37], [38], [39], [40], [41]. Based on this analysis, the residues were identified as follows: T-α-Araf-(1→, →6)-β-Galp-(1→, →3,6)-β-Galp-(1→, T-β-Rhap-(1→, →2,4)-α-Rhap-(1→, →4)-α-D-Glcp-(1→, →4)-β-D-Xylp-(1→, →6)-α-D-Manp-(1→, →5)-α-L-Araf-(1→, and → 3)-α-D-Manp-(1→, respectively (Table 5).

**Table 5:** ^1^H and ^13^C NMR chemical shifts of NATP-60a recorded in D_2_O.

Sugar residue	Chemical shifts (ppm)					
	C1/ H1	C2/H2	C3/H3	C4/H4	C5/H5	C6/H6
A T-α-Araf- (1→	107.47	76.54	81.31	83.83	61.08	
	5.10	3.95	4.14	4.08	3.73	
B → 6)-β-Galp-(1→	103.39	70.69	73.36	70.23	76.54	68.60
	4.49	3.67	3.98	3.77	3.67	4.14
C → 3,6)-β-Galp-(1→	103.68	70.23	80.31	76.54	73.27	72.56
	4.50	3.55	3.73	4.05	3.83	3.67
D T-β-Rhap- (1→	96.30	73.37	70.00	73.61	72.05	16.39
	4.65	3.51	3.85	3.55	4.07	1.25
E → 2,4)- α-Rhap −(1→	98.19	78.32	68.60	80.31	71.89	16.79
	5.20	4.19	3.95	3.74	3.86	1.33
F → 4)- α-D-Glcp-(1→	97.53	72.56	76.69	75.56	73.65	62.72
	5.34	3.27	3.78	3.52	3.44	3.82
G → 4)-β-D-Xylp-(1→	100.71	71.02	73.65	80.31	66.46	
	4.74	3.75	3.69	3.23	3.76	
H → 6)-α-D-Manp-(1→	100.07	70.13	70.23	67.60	70.69	65.65
	5.29	4.13	3.78	3.51	4.05	4.14
I → 5)-α-L-Araf −(1→	109.24	81.25	76.22	76.54	73.54	
	5.25	4.23	3.80	4.07	3.37	
J → 3)- α-D- Manp −(1→	101.56	71.89	68.50	64.75	62.72	
	5.05	3.95	3.69	3.77	3.82	

As illustrated in Fig. 5C, the linkage between residue F and E was identified by the correlation signal at 5.34/78.32 ppm (F H1/E C2), indicating that residue F is terminated by E at the C-2 position. Similarly, the connection between residue H and C was detected at 5.20/72.56 ppm (H H1/C C6), confirming residue H linked to C at the C-6 position. A signal at 5.05/65.65 ppm (J H1/H C6) revealed the linkage between residue J and H, suggesting termination of J at the C-6 position of H. The correlation observed at 4.74/68.60 ppm (G H1/B C6) indicated that residue G is connected to B at the C-6 position.

Furthermore, the association between residue D and J was supported by the signal at 4.65/68.50 ppm (D H1/J C3), implying that D terminates at the C-3 position of J. The interaction between residue E and C was further evidenced by a signal at 5.20/80.31 ppm (E H1/C C3), indicating linkage at the C-3 position. Additionally, a signal at 5.25/80.31 ppm (I H1/G C4) suggested that residue I is attached to G at the C-4 position. Conversely, the correlation at 3.40/103.68 ppm (C C1/I H5) indicated that residue I is linked to C at the C-1 position. Finally, a signal at 3.52/107.47 ppm (A C1/F H4) established that residue F is terminated by A at the C-1 position. The repeating unit of NATP-60a was elucidated (Fig. 5D) based on comprehensive analysis using 1D and 2D NMR spectroscopy in conjunction with complementary structural characterization techniques.

### 3.7 Morphological analysis of NATP-60a

**Fig. 6:**
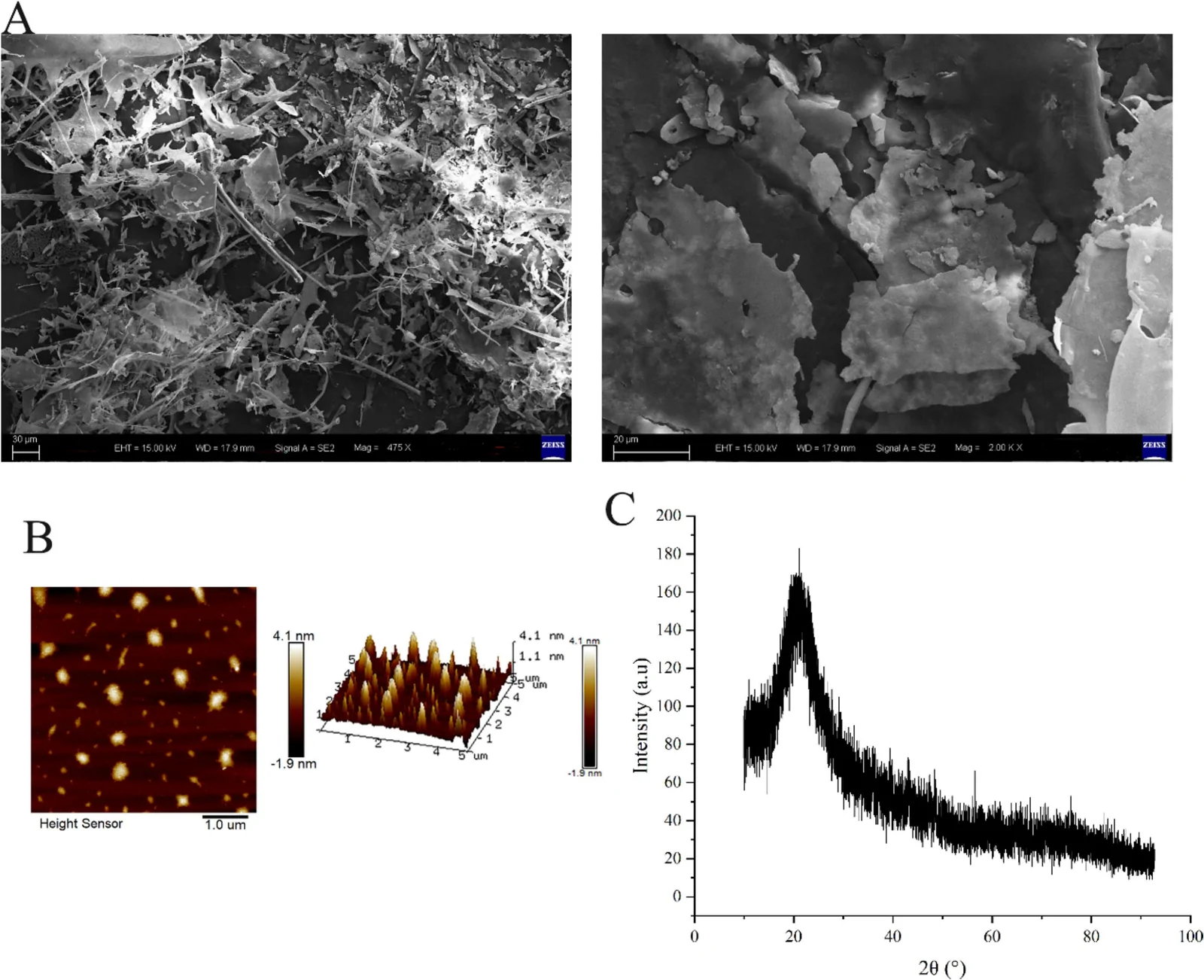
Morphological analysis and physicochemical properties of NATP-60a. A: scanning electron microscopy images (475×, 2000× ); B: AFM images; C: XRD analysis.

Polysaccharides from diverse sources exhibit considerable variation in their morphological structures, which significantly influences their biological activities. Here, we characterized the morphological features of NATP-60a. As shown in Fig. 6A, NATP-60a exhibits a flake-like morphology, characterized by a porous and loosely organized structure. As shown in Fig. 6B, NATP-60a primarily consists of irregularly shaped particulate aggregates, with a measured molecular height of approximately 4.1 nm. Furthermore, the XRD pattern of NATP-60a (Fig. 6C) displays a broad diffraction halo centered at 2θ = 20.15°, indicating a predominantly amorphous structure with substantial disordered regions [42].

### 3.8 NATP-60a alleviates DSS-induced colitis in mice

**Fig. 7:**
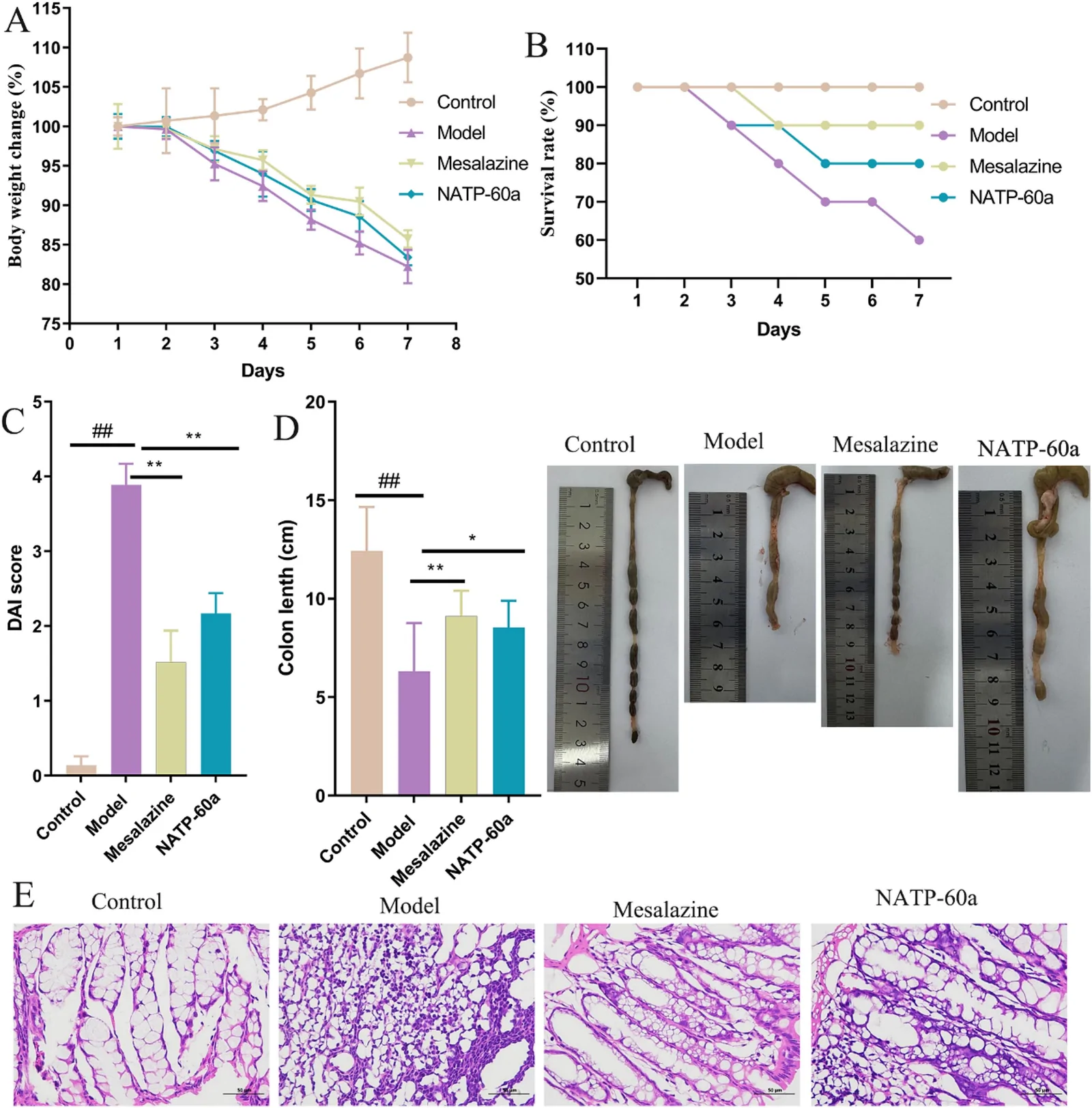
Effect of NATP-60a supplementation on the symptoms of DSS-induced colitis. (A) Changes in mice body weight. (B) Survival rate. (C) DAI score. (D) Changes in colon length and colon photographs. (E) Colonic tissue morphology. Data are presented as means ± SE, n = 8. ^##^p < 0.01 compared to the Control group. ** and * p < 0.01, p < 0.05 compared to the Model group.

We established DSS-induced colitis in mice to explore the therapeutic effect of NATP-60a. As illustrated in Fig. 7A, DSS administration resulted in significant body weight loss, which was ameliorated by both NATP-60a and mesalazine (YC) treatment. Furthermore, DSS increased the mortality rate, while both NATP-60a and YC effectively reduced mortality (Fig. 7B). DSS also markedly elevated the disease activity index (DAI), and this increase was attenuated by administration of NATP-60a or YC (Fig. 7C). Colon length was assessed across all four experimental groups, showing changes consistent with those observed in body weight and DAI scores (Fig. 7D). Histological examination via H&E staining revealed severe mucosal damage, partial crypt loss, and reduction of goblet cells in DSS-treated mice. Supplementation with NATP-60a notably reduced inflammatory infiltration and mitigated epithelial erosion in colonic tissues (Fig. 7E).

**Fig. 8:**
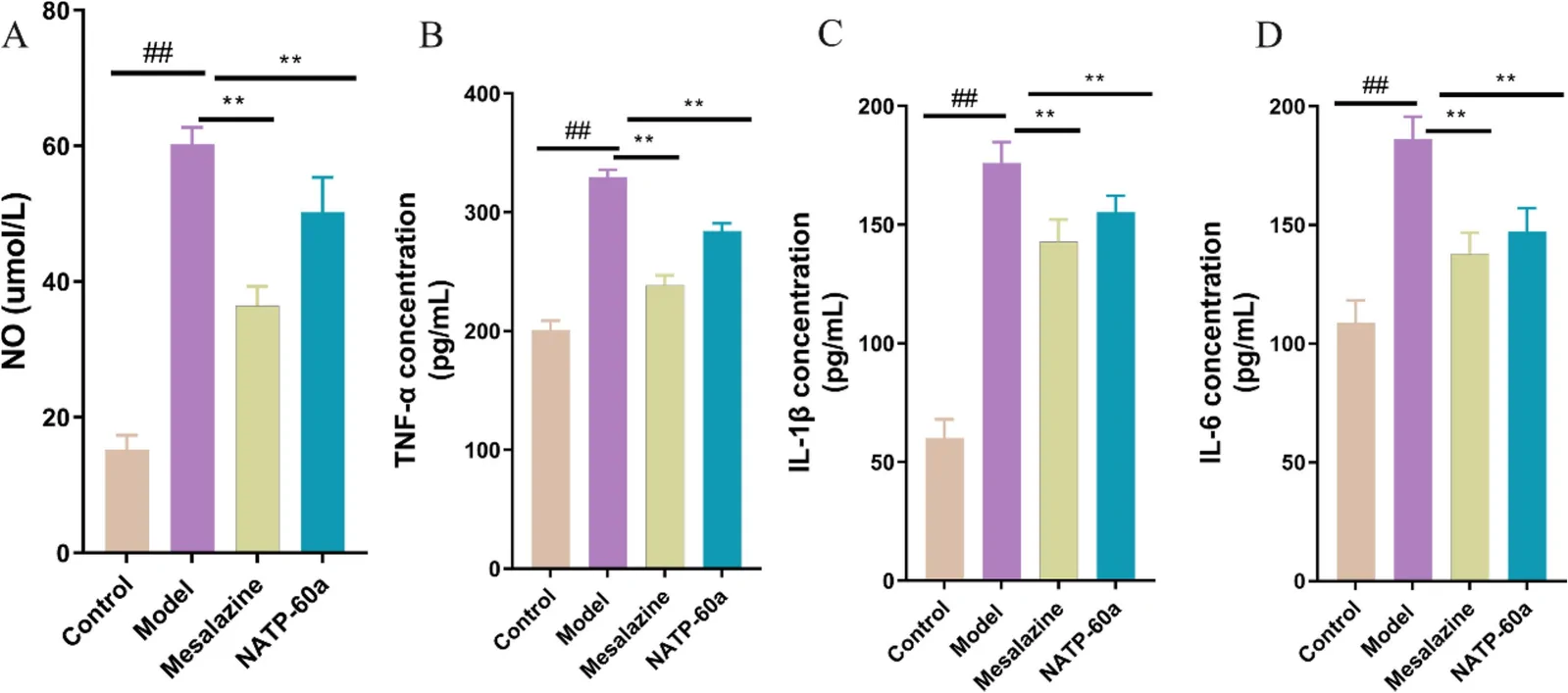
NATP-60a inhibits the production of inflammatory mediators and cytokines. (A) NO, (B) TNF-α, (C) IL-1β and (D) IL-6. Data are presented as means ± SE, n = 8. ## p < 0.01 compared to the Control group. ** and * *p* < 0.01, *p* < 0.05 compared to the Model group.

We assessed alterations in inflammatory factor levels. In comparison with the control group (CK), the model group (MC) demonstrated a marked rise in nitric oxide (NO) concentration (*p* < 0.01). In contrast, administration of NATP-60a significantly attenuated NO levels relative to the MC group (*p* < 0.01) (Fig. 8A). Furthermore, DSS administration elevated the concentrations of pro-inflammatory cytokines (*p* < 0.01). Notably, the changes in these markers following NATP-60a supplementation mirrored those observed with the positive control treatment (Fig. 8B–D), indicating that NATP-60a confers a protective effect against colitis. Together, these results demonstrate that NATP-60a effectively alleviates symptoms of DSS-induced colitis.

### 3.9 NATP-60a affects SCFAs content in colitis mice

**Fig. 9:**
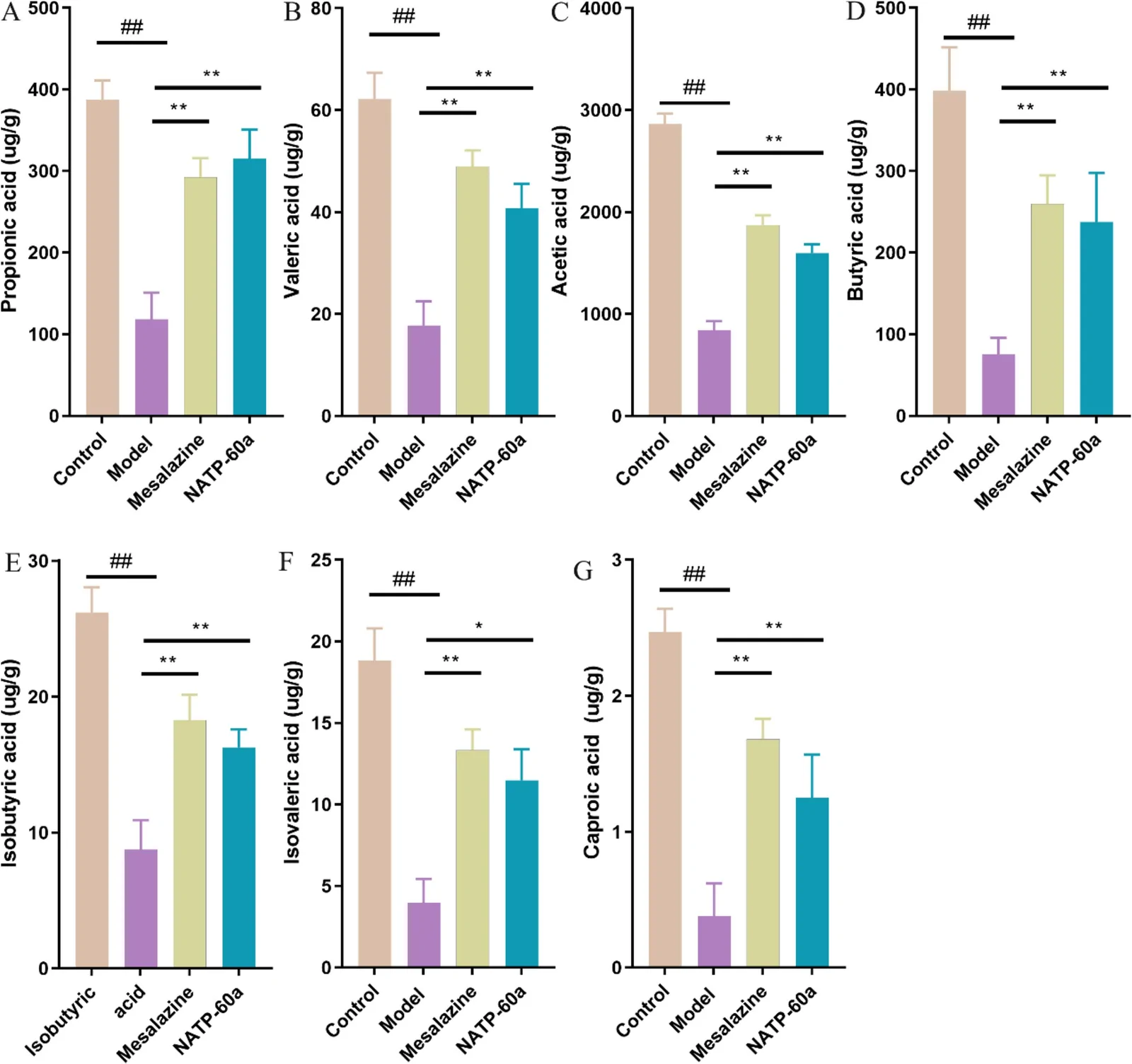
Effects of NATP-60a on production of SCFAs. (A) Propionic acid, (B) Valeric acid, (C) Acetic acid, (D) Butyric acid, (E) Isobutyric acid, (F) Isovaleric acid, (G) Caproic acid. Data are presented as means ± SE, n = 8. ^##^ and ^#^*p* < 0.05, *p* < 0.01 compared to the Control group. ** and * *p* < 0.05, *p* < 0.01 compared to the Model group.

SCFAs are primary metabolites produced by gut microbial fermentation of dietary polysaccharides, and exert critical effects on sustaining intestinal homeostasis. Here, we quantified fecal SCFAs in mice—including acetic acid, propionic acid, butyric acid, isobutyric acid, isovaleric acid, valeric acid, and caproic acid—using GC–MS. Our results revealed a significant reduction in SCFA levels in the MC group compared to the CK group (Fig. 9). In contrast, both the NATP-60a and YC groups exhibited a marked increase in SCFA concentrations relative to the MC group (Fig. 9). These results indicate that NATP-60a can relieve DSS-triggered colitis in mice by regulating intestinal microbial structure and boosting SCFA synthesis.

### 3.10 NATP-60a improves the intestinal microbiota in mice with colitis

**Fig. 10:**
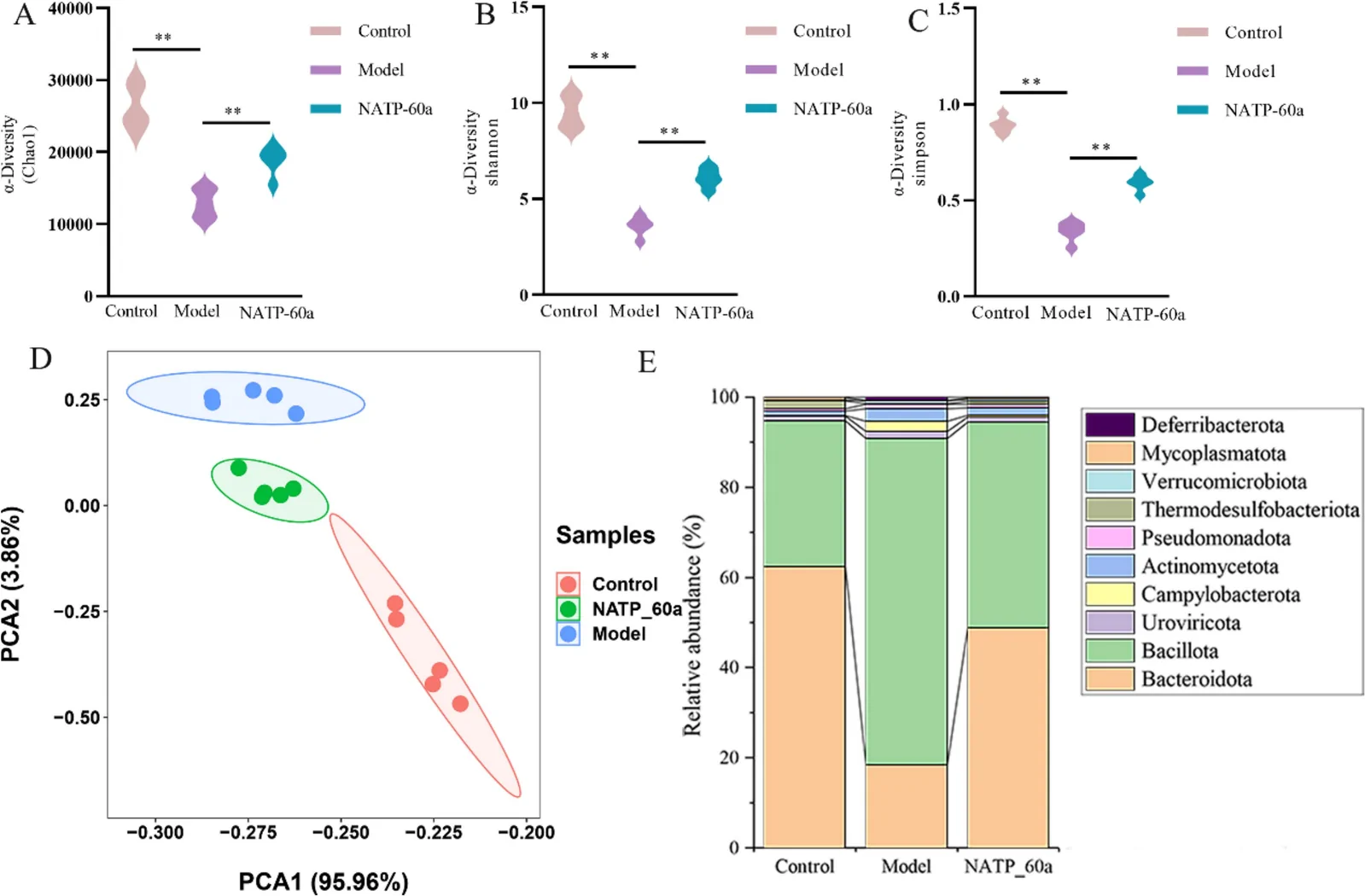
Effects of NATP-60a on the gut microbiota of DSS-induced colitis mice. The alpha diversity of gut microbiota estimated by Chao1 (A), Shannon (B), and Simpson evenness (C) indexes. The beta diversity of gut microbiota estimated by PCoA analysis on genus level (D). Bar plot of microbiota community composition at phylum (E). Significance level: *p < 0.05,^**^p < 0.01.

Gut microbial imbalance is a major contributor to colitis onset. To explore how NATP-60a relieves DSS-triggered colitis in mice, 16S rRNA gene sequencing was adopted to analyze microbial variations in fecal samples. We assessed gut microbial α-diversity based on Chao1, Shannon and Simpson indices. Compared with the CK, the DSS-induced model group exhibited significant reductions in all three indices. Notably, administration of NATP-60a significantly restored these values (Fig. 10A–C). These findings suggest that DSS disrupts gut microbial balance, whereas NATP-60a appears to promote homeostasis by enhancing microbial richness, diversity, and evenness. Beyond within-sample (α) diversity, we examined differences in microbial community structure (β-diversity) via principal coordinate analysis PCoA (Fig. 10D). This analysis demonstrated a clear separation among groups, with the NATP-60a group exhibiting a microbial profile that was intermediate between the control and model groups. This structural shift suggests that NATP-60a treatment effectively modulates the overall composition of the gut microbiota in colitis mice. Analysis of the predominant bacterial phyla revealed that the dysbiosis characterized by a decreased Bacteroidota/Bacillota ratio induced by DSS challenge was notably counteracted by NATP-60a (Fig. 10E). The ability of NATP-60a to reverse this specific phylum-level imbalance further supports its role in mitigating colitis-associated microbial dysbiosis.

**Fig. 11:**
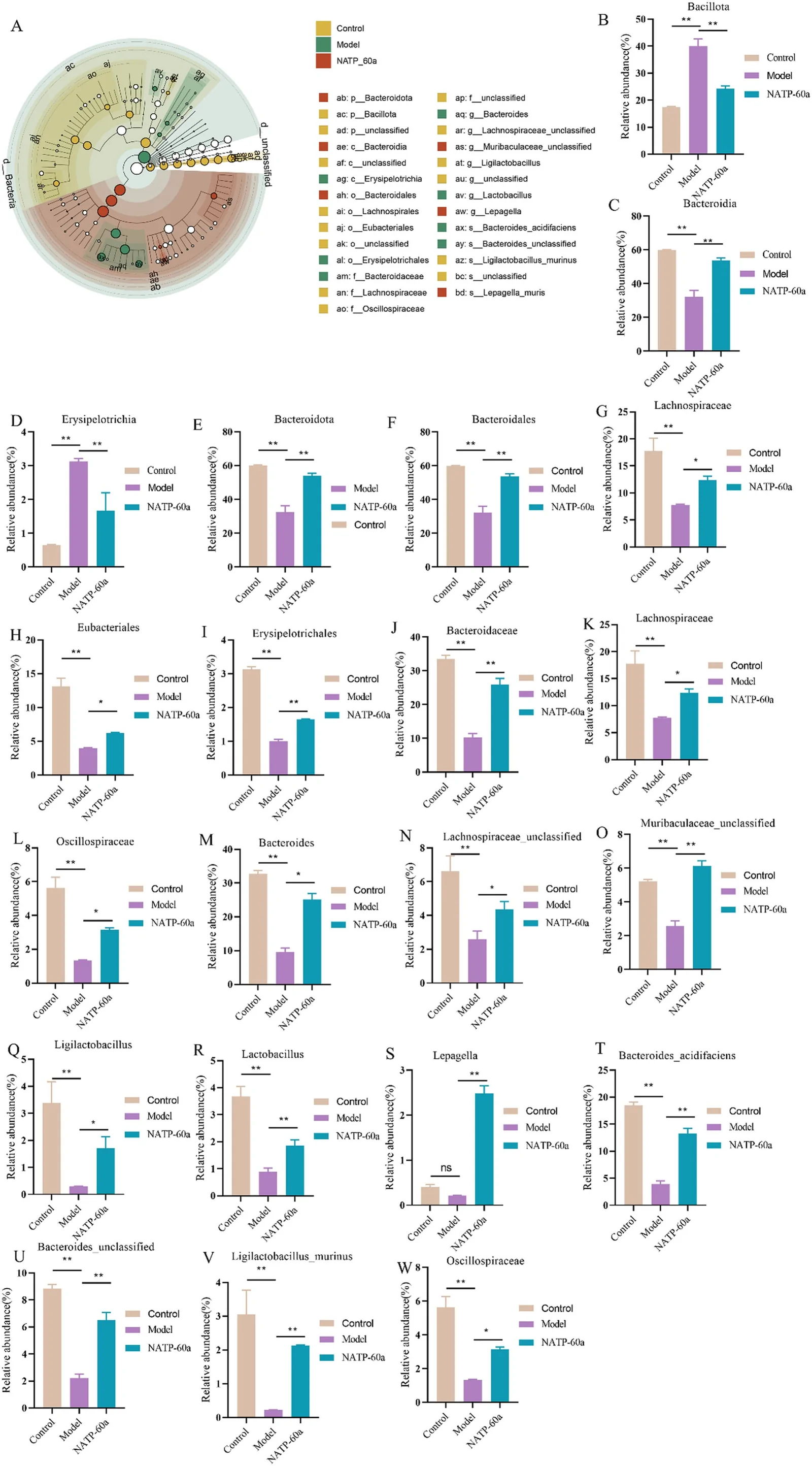
LEfSe analysis at different species levels (A). Changes in abundance of significantly altered species (B-W). Significance level: *p < 0.05, **p < 0.01, ns: no significance.

We then employed LDA Effect Size (LEfSe) analysis to identify bacterial taxa associated with colitis pathogenesis (Fig. 11A). Significant differences in community structure were observed among the groups: Bacillota, Lachnospirales, Eubacteriales, Lachnospiraceae, Oscillospiraceae, Lachnospiraceae_unclassified, and *Ligilactobacillus* showed notable variation under baseline conditions. DSS treatment induced marked shifts in the abundances of Erysipelotrichia, Erysipelotrichales, Bacteroidaceae, *Bacteroides*, and *Lactobacillus*. Administration of NATP-60a, in turn, led to significant changes in Bacteroidota, Bacteroidia, Bacteroidales, Muribaculaceae, *Lepagella*, and *Lepagella_muris*.

To further elucidate the potential regulatory mechanism of NATP-60a, we examined the detailed dynamics of these bacterial communities (Fig. 11B–W). Our results revealed that DSS treatment significantly reduced the abundance of multiple SCFA-producing bacteria. Notably, supplementation with NATP-60a restored the levels of several taxa, including Bacteroidota, Bacteroidia, Bacteroidales, Muribaculaceae, *Lepagella, Lepagella_muris, Ligilactobacillus,* and Lachnospiraceae_unclassified. This restoration of SCFA-producing bacteria may represent a key mechanism through which NATP-60a alleviates colitis.

### 3.11 NATP-60a reverses the metabolic profile of colitis in mice

**Fig. 12:**
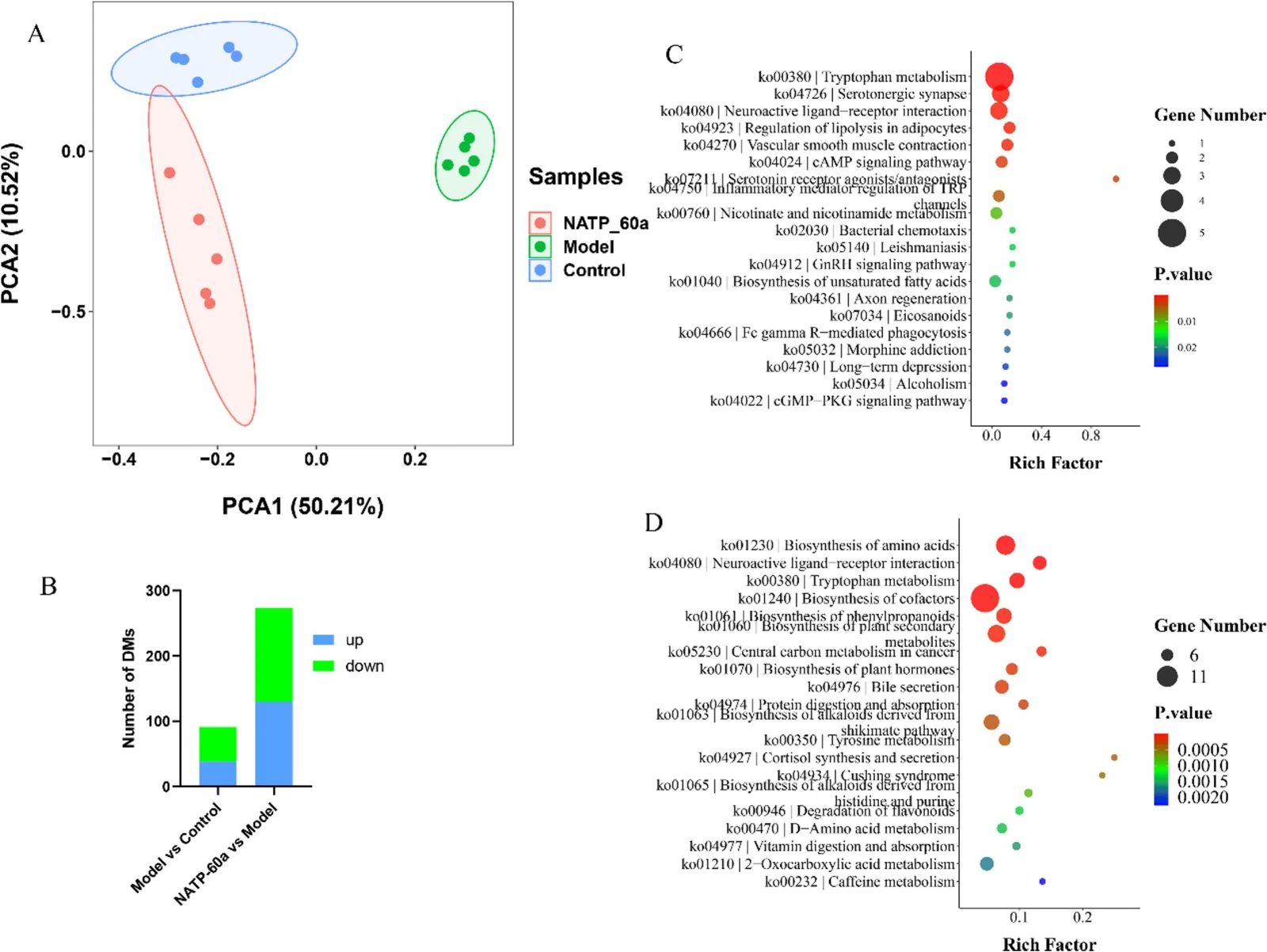
Metabolomic analysis of mouse intestinal contents.

We next performed metabolomic profiling of mouse intestinal contents. Principal component analysis (PCA) showed distinct grouping differences across control, model and NATP-60a-administered groups, suggesting that DSS induction and NATP-60a intervention remarkably modified intestinal metabolic characteristics (Fig. 12A). According to partial least squares-discriminant analysis (PLS-DA), metabolites satisfying the thresholds of fold change (FC) ＞1.2, variable importance in projection (VIP)＞1 and p＜0.05 were identified as differential metabolites (DMs). In the Model vs. Control comparison, 91 DMs were identified, of which 38 were upregulated and 53 were downregulated. In the NATP-60a vs. Model comparison, 273 DMs were identified, including 130 upregulated and 143 downregulated (Fig. 12B). We observed that across all comparisons, the number of downregulated metabolites exceeded that of upregulated ones; however, NATP-60a treatment resulted in a greater number of upregulated metabolites than observed in the DSS model group, suggesting that DSS may reduce overall metabolic activity in mice, whereas NATP-60a may alleviate colitis by enhancing metabolic flux.

KEGG pathway annotation of the identified DMs showed that the 91 DMs from the Model vs. Control comparison were mapped to 91 metabolic pathways, among which 33 pathways were significantly altered (*p* < 0.05). The 273 DMs from the NATP-60a vs. Model comparison were also mapped to 91 pathways, with 55 being significantly altered (*p* < 0.05). We further analyzed the top 20 most significantly altered pathways in each group. The results indicated that DSS markedly affected tryptophan metabolism, serotonergic synapse, neuroactive ligand-receptor interaction, regulation of lipolysis in adipocytes, vascular smooth muscle contraction, cAMP signaling pathway, serotonin receptor agonists/antagonists, inflammatory mediator regulation of TRP channels, nicotinate and nicotinamide metabolism, GnRH signaling pathway, leishmaniasis, bacterial chemotaxis, and biosynthesis of unsaturated fatty acids (Fig. 12C). In contrast, NATP-60a ameliorated DSS-induced colitis by modulating the biosynthesis of amino acids, neuroactive ligand-receptor interaction, tryptophan metabolism, biosynthesis of cofactors, biosynthesis of phenylpropanoids, central carbon metabolism in cancer, bile secretion, protein digestion and absorption, biosynthesis of alkaloids derived from the shikimate pathway, and tyrosine metabolism (Fig. 12D).

**Fig. 13:**
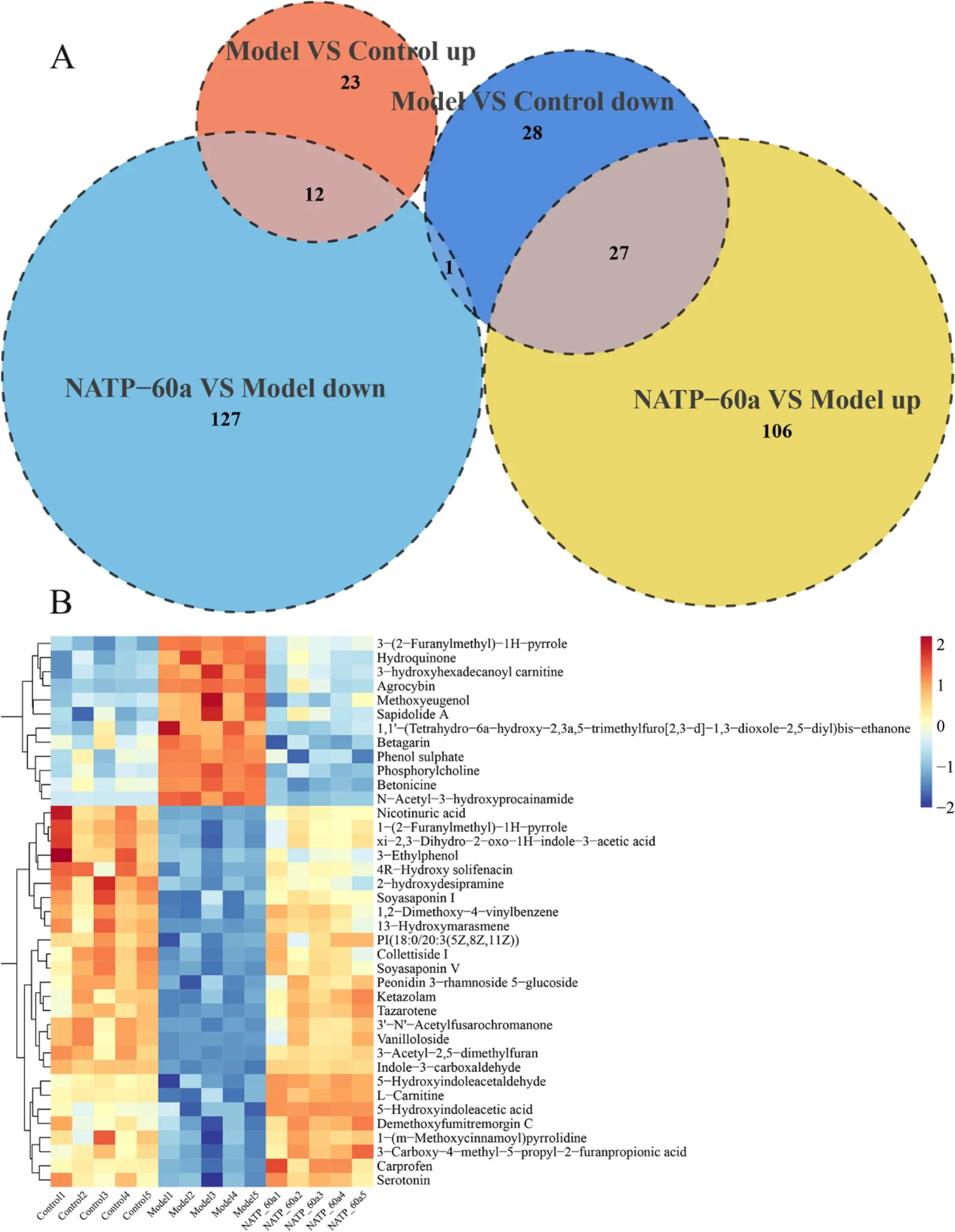
NATP-60a reversed the metabolic profile of colitis mice.

To further investigate the mechanism by which NATP-60a ameliorates colitis, we conducted an in-depth analysis of the DMs across different groups. We defined as key metabolites those that showed opposite trends in abundance after DSS treatment and subsequent NATP-60a intervention, suggesting their potential role in the amelioration of colitis (Fig. 13). Venn diagram analysis revealed that among the metabolites downregulated by DSS, 27 were upregulated after NATP-60a treatment. Conversely, among those upregulated by DSS, 12 were downregulated following NATP-60a administration. Detailed analysis of the relative abundance changes of these key compounds highlighted several metabolites, including 3-(2-Furanylmethyl)-1H-pyrrole, Sapidolide A, Betonicine, Betagarin, Nicotinuric acid, among others.

### 3.12 Correlation between intestinal microbial species and differential metabolites

**Fig. 14:**
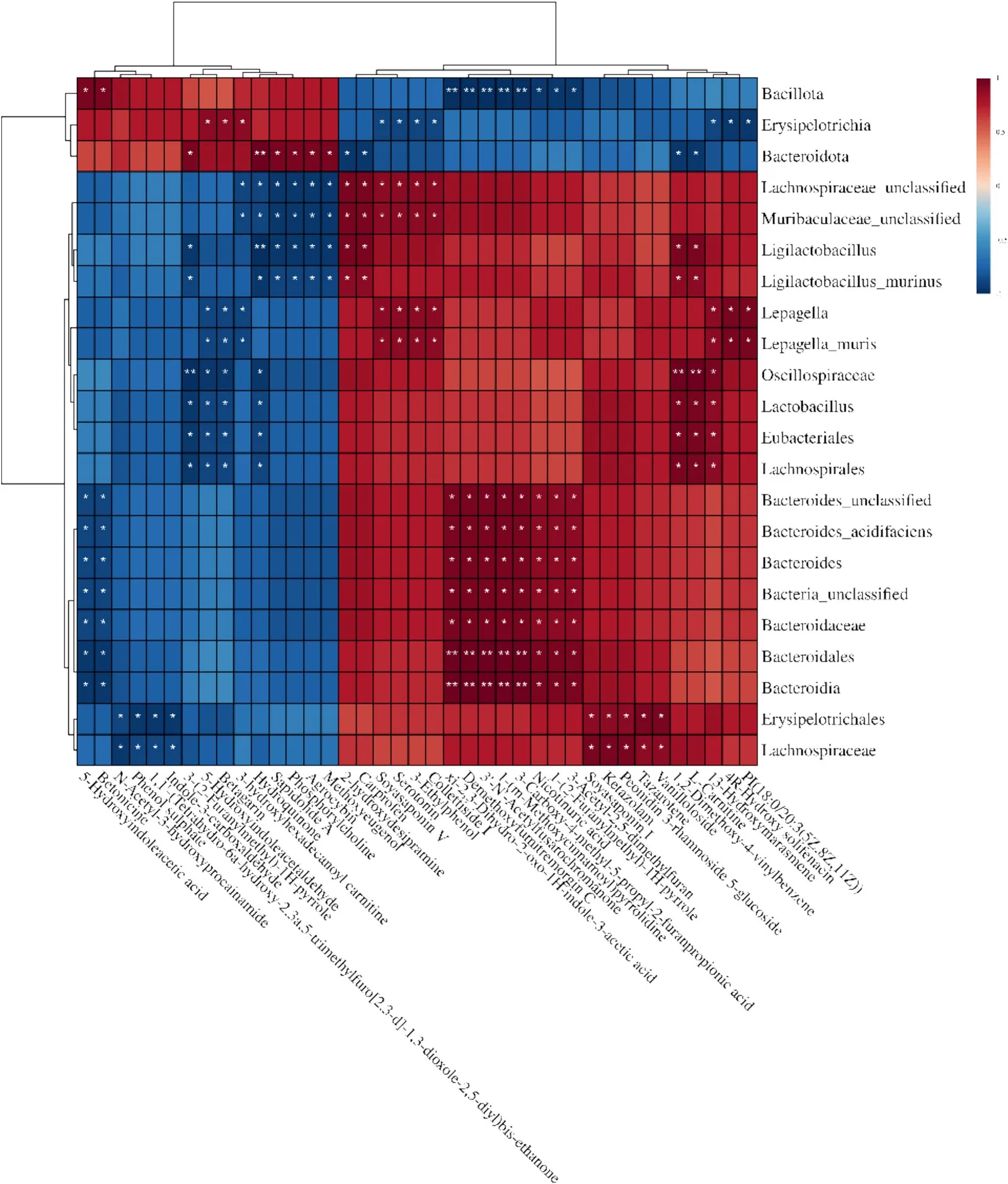
Correlation analysis between key differential metabolites and bacterial abundance.

To further elucidate the interactions between the 39 key differential metabolites and gut microbiota, Spearman's correlation analysis was conducted to assess their associations (Fig. 14). The results revealed strong negative correlations (*p* < 0.01) between Bacillota and several metabolites, including 1-(m-Methoxycinnamoyl) pyrrolidine, 3′-N'-Acetylfusarochromanone, 3-carboxy-4-methyl-5-propyl-2-furanpropionic acid, demethoxyfumitremorgin C, xi-2,3-dihydro-2-oxo-1H-indole-3-acetic acid, hydroquinone, and 3-(2-furanylmethyl)-1H-pyrrole. In contrast, Bacteroidales and Bacteroidia exhibited strong positive correlations (*p* < 0.01) with metabolites such as 1-(m-Methoxycinnamoyl) pyrrolidine, 3′-N'-Acetylfusarochromanone, 3-carboxy-4-methyl-5-propyl-2-furanpropionic acid, demethoxyfumitremorgin C, and xi-2,3-dihydro-2-oxo-1H-indole-3-acetic acid. Additionally, Oscillospiraceae showed significant positive correlations (*p* < 0.01) with 1,2-dimethoxy-4-vinylbenzene and L-carnitine. These findings suggest that 1-(m-Methoxycinnamoyl) pyrrolidine, 3′-N'-Acetylfusarochromanone, 3-carboxy-4-methyl-5-propyl-2-furanpropionic acid, demethoxyfumitremorgin C, and xi-2,3-dihydro-2-oxo-1H-indole-3-acetic acid may represent key metabolites through which NATP-60a modulates gut microbiota abundance.

## 4 Discussion

Recently, natural plant polysaccharides have been found to exhibit diverse pharmacological effects against diabetes, inflammation and cancer, with advantages of safety, high efficacy, non-toxicity, low cost and good biocompatibility. Furthermore, plant polysaccharides possess good anti-inflammatory and immunomodulatory activities, showing a significant and direct effect on colitis [43]. This study purified a novel polysaccharide fraction, NATP-60a, from the unique ancient tree of Nanchuan in southwestern China. This fragment was able to improve colitis in mice, and its structural characteristics were studied in detail. The results show that NATP-60a can significantly inhibit cellular inflammation and improve colonic function by regulating the concentration of intestinal flora and key metabolites.

In this study, we used DSS to establish a mouse model of acute colitis and intervened with NATP-60a. Previous studies have demonstrated that DSS administration induces typical colitis symptoms in mice, including diarrhea, bloody stools, weight loss, shortened colon length and increased DAI scores [44]. As anticipated, administration of DSS successfully induced colitis in mice, as evidenced by the characteristic symptoms presented in Fig. 7. Notably, intervention with NATP-60a significantly ameliorated DSS-induced clinical manifestations, including reduced fecal bleeding, increased colon length, and decreased DAI scores. These findings are consistent with previous reports demonstrating that polysaccharides derived from *Phyllanthus emblica L*. alleviate body weight loss, improve DAI scores, and mitigate colonic pathological damage in DSS-induced colitis models [45]. Collectively, our data suggest that NATP-60a plays a protective role in attenuating experimental colitis in mice.

Having established the anti-inflammatory properties of NATP-60a in LPS-induced RAW264.7 macrophages, as it mitigated pro-inflammatory cytokine release (Fig. 2), we then investigated its role in a DSS-induced colitis model—a condition pathologically associated with overproduction of inflammatory mediators and cytokines such as NO, IL-1β, IL-6, and TNF-α. In vivo outcomes were consistent with in vitro observations. NATP-60a intervention markedly lowered colon NO content, along with the levels of core inflammatory cytokines TNF-α, IL-1β and IL-6 in DSS-induced mice. These data demonstrate that the amelioration of colitis by NATP-60a is mediated through a concerted action of suppressing inflammatory responses and suppressing the inflammatory cascade.

SCFAs are major products of gut microbial polysaccharide fermentation, serving core functions in colonic wellness and immune modulation. Notably, SCFA levels are consistently depleted in patients with colitis and DSS-induced murine models [46]. Previous studies have shown that *Astragalus* polysaccharides alleviate DSS-induced colitis by enhancing the concentrations of specific SCFAs. Consistent with these findings, our study confirmed a significant reduction in colonic SCFAs (including propionic, valeric, acetic, butyric, isobutyric, isovaleric, and caproic acids) following DSS administration. Importantly, NATP-60a intervention effectively restored the levels of five key SCFAs (Fig. 9). Collectively, these results indicate that the restoration of SCFAs may represent a pivotal mechanism through which NATP-60a ameliorates experimental colitis.

Gut microbiota exerts a critical influence on intestinal homeostasis, and microbial imbalance is intimately correlated with the pathological progression of colitis. In murine models of colitis, microbial imbalance is characterized by altered metabolic outputs: Bacteroides ferment carbohydrates into SCFAs, such as butyrate, which is crucial for gut health, whereas Actinobacteria (including *Bifidobacterium*) contribute to intestinal barrier integrity [47]. Conversely, potential pathobionts such as *Pseudomonas* and *Fusobacterium* are associated with intestinal inflammation [48]. In this study, we employed 16S rRNA sequencing to investigate the impact of NATP-60a on the gut microbiota in DSS-induced colitis mice. Our analysis revealed a marked reduction in microbial diversity and a profound structural disruption of the microbiota in colitis mice. Notably, NATP-60a treatment modulated the microbial composition, shifting it closer to that of the control group (Fig. 10). Further examination at the phylum level demonstrated that DSS administration increased the abundance of Bacillota and decreased that of Bacteroidota. NATP-60a intervention reversed these alterations, a phenomenon similarly observed with polysaccharides from *Dioscorea* and *Polygonatum cyrtonema* Hua [49], [50]. Using LEfSe analysis, we identified taxa most significantly influenced by NATP-60a in colitic mice, including Bacteroidota, Bacteroidia, Bacteroidales, Muribaculaceae, *Lepagella*, *Lepagella_muris*, *Ligilactobacillus*, and unclassified Lachnospiraceae (Fig. 11). Detailed assessment of their abundance changes revealed that members of the Bacteroidetes phylum, specifically Bacteroidota, Bacteroidia, and Bacteroidales, were prominently modulated by NATP-60a. Recent evidence indicates that Bacteroidetes populations, capable of utilizing diverse polysaccharides, remain stable throughout the human lifespan and across generations [51], and play a significant role in colitis and immune dysfunction [52]. Consistent with our findings, *Poria cocos* polysaccharide CMP I alleviates DSS-induced colitis by enriching Bacteroidales [53]; Bacteroidia enhances gut barrier function by fermenting *Cantharellus cibarius* Fr. polysaccharides in DSS-treated mice [54]; and Bacteroidota improves intestinal barrier integrity through encoding abundant carbohydrate-active enzymes that facilitate polysaccharide hydrolysis [55]. Muribaculaceae, a beneficial family, modulates gut microbiota composition and ameliorates inflammation in DSS-induced colitis [56]. As a butyrate producer, it degrades complex carbohydrates, promotes mucosal layer formation, and strengthens the gut barrier, thereby playing a key role in maintaining gastrointestinal homeostasis [57]. Within this family, *Lepagella* and *Lepagella_muris* were identified as major characteristic species [57], their abundance increased upon NATP-60a supplementation (Fig. 11). Similarly, *Ligilactobacillus*, a beneficial bacterium that promotes SCFA production, alleviates immune checkpoint blocker-induced colitis via activation of the colonic GPR109A pathway [58]. Lachnospiraceae also attenuates DSS-induced colitis in mice by elevating levels of acetate, propionate, and butyrate [59]. Collectively, these results suggest that NATP-60a alleviates intestinal inflammation in colitis mice primarily by remodeling the gut microbiota and enhancing SCFA production.

Metabolites derived from the gut microbiota serve as key molecular mediators in host–microbiota interactions, exerting diverse and critical effects on host physiology. In this study, we performed untargeted metabolomics to profile fecal metabolites in a DSS-induced colitis mouse model, aiming to elucidate the impact of NATP-60a on gut microbial metabolism. Following DSS administration, the levels of several metabolites were significantly elevated, including 1,1′-(Tetrahydro-6a-hydroxy-2,3a,5-trimethylfuro[2,3–d]-1,3-dioxole-2,5-diyl)bis-ethanone, 3-(2-Furanylmethyl)-1H-pyrrole, 3-hydroxyhexadecanoyl carnitine, agrocybin, betagarin, betonicine, hydroquinone, N-Acetyl-3-hydroxyprocainamide, phenol sulphate, phosphorylcholine, and sapidolide A. Conversely, a number of metabolites exhibited marked reductions, such as 1,2-Dimethoxy-4-vinylbenzene, 1-(2-Furanylmethyl)-1H-pyrrole, 1-(m-Methoxycinnamoyl)pyrrolidine, 13-Hydroxymarasmene, 2-hydroxydesipramine, 3′-N'-Acetylfusarochromanone, 3-Acetyl-2,5-dimethylfuran, 3-Carboxy-4-methyl-5-propyl-2-furanpropionic acid, 3-Ethylphenol, 4R-Hydroxy solifenacin, Carprofen, Collettiside I, Demethoxyfumitremorgin C, Ketazolam, L-Carnitine, Nicotinuric acid, PI(18:0/20:3(5Z,8Z,11Z)), Peonidin 3-rhamnoside 5-glucoside, Serotonin, Soyasaponin I, Soyasaponin V, Tazarotene, Vanilloloside, xi-2,3-Dihydro-2-oxo-1H-indole-3-acetic acid, Indole-3-carboxaldehyde, Methoxyeugenol, 5-Hydroxyindoleacetaldehyde, and 5-Hydroxyindoleacetic acid (Fig. 13). Notably, NATP-60a intervention reversed these alterations in metabolite abundance. Previous studies have reported that 5-hydroxyindoleacetaldehyde and 5-hydroxyindoleacetic acid, as key intermediates in tryptophan metabolism, participate in the disease process in DSS-induced colitis models [60], [61]. In addition, indole-3-carboxaldehyde can reduce the expression of inflammatory factors and repair the intestinal barrier function in mice with ulcerative colitis by inhibiting the TLR4/NF-κB/MAPK p38 signaling pathway [62]. Methoxyeugenol can activate PPAR-α, thereby inhibiting NF-κB-mediated inflammatory signaling and oxidative stress response, thus alleviating colitis [63]. The metabolite changes observed in this study are consistent with these mechanisms, suggesting that NATP-60a may improve metabolic disorders and exert a therapeutic effect in colitis mice by regulating the levels of these key metabolites.

Additionally, following NATP-60a administration, we detected significant modulations in a range of metabolic pathways, including amino acid metabolism, tryptophan metabolism, tyrosine metabolism, cofactor biosynthesis, phenylpropanoid biosynthesis, bile secretion, and protein digestion and absorption. These pathways have been reported as key metabolic pathways distinguishing colitis patients from healthy individuals, suggesting their potential involvement in disease development. Prior investigations confirm amino acids are crucial components for maintaining the structural integrity and barrier capability of intestinal tissues [64], and tryptophan deficiency, in particular, significantly exacerbates colitis pathological damage [65]. In the gut, the microbiota regulates three major pathways of tryptophan metabolism directly or indirectly: serotonin (5-hydroxytryptamine) synthesis, the kynurenine pathway, and indole derivative generation. Metabolites derived from tryptophan can stimulate aryl hydrocarbon receptor (AhR), a critical modulator of innate immunity [66]. Its activation can promote regulatory T cell differentiation, inhibit Th17 and Th1 cell responses, and induce the production of anti-inflammatory mediators [67]. Studies have confirmed that *Ascophyllum nodosum* polysaccharide may exert anti-inflammatory effects by regulating tyrosine metabolism [68]. Furthermore, bergamot polysaccharides have also been shown to alleviate DSS-induced colitis in mice by regulating tryptophan and tyrosine metabolism [69]. These findings collectively suggest that NATP-60a may exert its protective effect against colitis by coordinating multiple key metabolic pathways, particularly amino acid and related metabolic networks. Notably, we propose that NATP-60a alleviates metabolic disorders predominantly via gut microbiota remodeling. As an 8.4 kDa macromolecule with low gastrointestinal absorption, it is mainly fermented by colonic microbiota. This is supported by restored SCFA-producing bacteria, recovered SCFA levels, and strong metabolite-microbiota correlations. Direct immunomodulatory effects on host cells cannot be fully excluded, but the microbiota-metabolite axis plays a dominant role based on current evidence.

## 5 Conclusion

In this study, the UAE process of NATP was first optimized, achieving an extraction yield of 3.84 ± 0.15 % under optimal conditions. Subsequently, a novel polysaccharide fraction designated as NATP-60a was isolated and purified, with a molecular weight of 8.4 kDa. Its monosaccharide composition consists mainly of Rha, Ara, Gal, Glc, Xyl, and Man. 16S rRNA analysis revealed that NATP-60a ameliorated colitis in mice by modulating the abundance of specific gut microbiota, such as Muribaculaceae, *Lepagella, Ligilactobacillus,* and unclassified Lachnospiraceae. Metabolomic findings further indicated that NATP-60a alleviated the progression of colitis mainly through regulating several metabolic pathways, including amino acid metabolism, tryptophan metabolism, tyrosine metabolism, biosynthesis of cofactors, and phenylpropanoid biosynthesis. In conclusion, our results demonstrate that NATP-60a possesses the capacity to mitigate DSS-induced colitis in mice and may serve as a promising prebiotic agent to restore gut microbial homeostasis and prevent colitis. Future work will validate the causal role of gut microbiota via antibiotic depletion and fecal microbiota transplantation.

## CRediT authorship contribution statement

**Deyong Zeng:** Writing – review & editing, Writing – original draft, Resources, Funding acquisition, Data curation. **Jianlong Wang:** Writing – review & editing, Software, Methodology, Formal analysis. **Junli Liu:** Visualization, Validation, Methodology. **Bichun Hu:** Validation, Software, Investigation. **Weiping Wang:** Visualization, Validation. **Shuaimin Hu:** Software, Project administration. **Cuihong Dai:** Visualization, Validation, Investigation. **Haitian Zhao:** Visualization, Validation. **Weihong Lu:** Supervision, Resources, Funding acquisition.

## Funding

This study was supported by the Natural Science Foundation of Chongqing, China (Grant No. CSTB2024NSCQ-MSX0863, CSTB2024NSCQ-MSX0462), the 2025 Employee Technical Innovation Project of the Chongqing Federation of Trade Unions (Grant No. KG517126001), and the Heilongjiang Touyan Team, China (Grant No. HITTY-20190034).

## Declaration of competing interest

The authors declare that they have no known competing financial interests or personal relationships that could have appeared to influence the work reported in this paper.

## Appendix A Supplementary data

Supplementary Data 1 UV–Vis and FT-IR spectra of NATP-60a.
