# Supplementary material for: Ultrasound-assisted extraction of novel ancient tea polysaccharide NATP-60a: Process optimization, structural characterization, and mechanism of alleviating DSS-induced colitis in mice
Source: Ultrason Sonochem. 2026 Aug 13;133:108005. doi: 10.1016/j.ultsonch.2026.108005 (PMC13524502; doi:10.1016/j.ultsonch.2026.108005)
Supplement: Supplementary file 1 [file mmc1.docx]

**Ultrasound-assisted extraction of novel ancient tea polysaccharide NATP-60a: process optimization, structural characterization, and mechanism of alleviating DSS-induced colitis in mice**

Deyong Zeng^a,b,c,d,1,^*, Jianlong Wang^a,b,d,1^, Junli Liu^a,b,c^ , Bichun Hu^a,b^, Weiping Wang^a,b^, Shuaimin Hu^a,b^, Cuihong Dai^a,b^, Haitian Zhao^a,c^, Weihong Lu^a,b,c,d,^*

*a Department of Food Science and Engineering, School of Medicine and Health, Harbin Institute of Technology, Harbin 150000, China*

*b National and Local Joint Engineering Laboratory for Synthesis, Transformation and Separation of Extreme Environmental Nutrients, Harbin Institute of Technology, Harbin, 150001, China*

*c The intelligent Equipment Research Center for the Development of Special Medicine and Food Resources, Chongqing Research Institute of HIT. Harbin Institute of Technology, Chongqing. 401120, China.*

*d Zhengzhou Advanced Research Institute of Harbin Institute of Technology, Zhengzhou 450003, China*

*^1^Co-first author:* Deyong Zeng, Jianlong Wang

** Corresponding author:*

Deyong Zeng*; Department of Food Science and Engineering, School of Medicine and Health, Harbin Institute of Technology; Harbin, Heilongjiang, China 150001; E-mail address: zengdy@hit.edu.cn;*

*Weihong Lu; Department of Food Science and Engineering, School of Medicine and Health, Harbin Institute of Technology; Harbin, Heilongjiang, China 150001; E-mail address: lwh@hit.edu.cn;*

Fig. S1. UV–Vis and FT-IR spectra of NATP-60a.(A) UV–Vis absorption spectrum; (B) FT-IR spectrum.
